# First finding of continental deep subduction in the Sesia Zone of the Western Alps and implications for subduction dynamics

**DOI:** 10.1093/nsr/nwad023

**Published:** 2023-01-20

**Authors:** Yi-Xiang Chen, Kun Zhou, Qiang He, Yong-Fei Zheng, Hans-Peter Schertl, Kun Chen

**Affiliations:** CAS Key Laboratory of Crust-Mantle Materials and Environments, School of Earth and Space Sciences, University of Science and Technology of China, Hefei 230026, China; Center of Excellence for Comparative Planetology, Chinese Academy of Sciences, Hefei 230026, China; CAS Key Laboratory of Crust-Mantle Materials and Environments, School of Earth and Space Sciences, University of Science and Technology of China, Hefei 230026, China; CAS Key Laboratory of Crust-Mantle Materials and Environments, School of Earth and Space Sciences, University of Science and Technology of China, Hefei 230026, China; CAS Key Laboratory of Crust-Mantle Materials and Environments, School of Earth and Space Sciences, University of Science and Technology of China, Hefei 230026, China; Center of Excellence for Comparative Planetology, Chinese Academy of Sciences, Hefei 230026, China; Institute of Geology, Mineralogy and Geophysics, Faculty of Geosciences, Ruhr University Bochum, Bochum 44780, Germany; CAS Key Laboratory of Crust-Mantle Materials and Environments, School of Earth and Space Sciences, University of Science and Technology of China, Hefei 230026, China

**Keywords:** coesite, ultrahigh-pressure metamorphism, continental deep subduction, Western Alps, far-field stress

## Abstract

Continental deep subduction after the closure of large oceanic basins is commonly ascribed to the gravitational pull of the subducting oceanic slab. However, it is not clear how continental lithosphere adjacent to small oceanic basins was subducted to mantle depths. The Sesia Zone in the Western Alps provides an excellent target for exploration of subduction dynamics in such a tectonic setting. Here we report the first finding of coesite in a jadeite-bearing orthogneiss from the Sesia Zone, providing the first evidence for deep subduction of the continental crust to mantle depths for ultrahigh-pressure (UHP) metamorphism in this zone. Three coesite inclusions were identified by laser Raman spectroscopy in two garnet grains. Based on zircon U-Pb dating and trace element analysis, the UHP metamorphic age was constrained to be 76.0 ± 1.0 Ma. The phase equilibrium modeling yields peak metamorphic pressures of 2.8–3.3 GPa, demonstrating the continental deep subduction to mantle depths of >80 km. The subducted continental crust was a rifted hyperextended continental margin, which was converted to the passive continental margin during seafloor spreading and then deeply subducted during the oblique convergence between the Adria microplate and Eurasian plate in the Late Cretaceous. Because the slab pull could only play a limited role in closing small oceanic basins for continental collision, the distal push of either continental breakup or seafloor spreading is suggested as the major driving force for the deep subduction of continental crust in the Western Alps. Therefore, deep subduction of the continental crust bordering small oceanic basins would have been induced by the far-field stress of compression, whereas that bordering large oceanic basins was spontaneous due to the oceanic slab pull. This provides a new insight into the geodynamic mechanism of continental deep subduction.

## Teaser

The ultrahigh-pressure metamorphism in the Sesia zone of Western Alps is firstly reported, demonstrating the deep subduction of continental crust to mantle depths of >80 km. The continental deep subduction is driven by the distal push of either continental breakup or seafloor spreading, rather than by the proximal pull of the subducting oceanic slab.

## INTRODUCTION

The finding of coesite in the continental crust has opened the new era of continental tectonics [1–3], demonstrating that low-density continental crust can be subducted to mantle depths for ultrahigh-pressure (UHP) metamorphism. It is generally agreed that the gravitational pull of the subducting oceanic slab serves as the driving force for the deep subduction of buoyant continental lithosphere to mantle depths of >80 km [4,5]. While this spontaneous mechanism is valid for deep subduction of the continental crust bordering large oceanic basins, it has been challenged by deep subduction of the continental crust bordering small oceanic basins [6]. This requires a resolution to the spatiotemporal relationships between the disappeared oceanic basin and the subsequently subducted continent, which are not always straightforward in collisional orogens [7]. Before continental collision, intercontinental basins may originally be small, embryonic or even absent. In this case, it is intriguing how continental subduction is initiated and which force drives the continental deep subduction to mantle depths [8].

The Western Alps is a typical locality for studying UHP metamorphism and continental deep subduction [9–11]. As a collisional orogen, its formation involves a series of episodic processes in the Tertiary from accretion of the oceanic crust through collision of the continental lithosphere, to exhumation of both oceanic and continental slices [7,12]. Among three petrotectonic units in the Western Alps, coesite was firstly discovered in the Dora-Maira Massif [1], where the UHP metamorphism was dated to ca. 35 Ma [13]. Subsequently, coesite and microdiamond were found in meta-ophiolite from the Lago di Cignana area of the Piemont-Liguria Zone [14,15], where the UHP metamorphism was dated to ca. 44 Ma [16]. So far no evidence of UHP metamorphism has been found in high-grade metamorphic rocks of the Sesia Zone, which is one NE-SW oriented unit ca. 25 by 100 km in size, mainly consisting of the Paleozoic continental basement [9,17]. Nevertheless, high-pressure (HP) metamorphic rocks are common in the Sesia Zone, leading to different tectonic models for their petrogenesis. These include tectonic erosion by the subducting Piemont-Liguria oceanic lithosphere [18], slab pull of an unknown oceanic lithosphere [19], tectonic burial without the pull of the subducting oceanic slab [20], and forced subduction by continental convergence between Adria and Europe [6].

Increasing field and petrological observations indicate that the Sesia Zone represents a hyperextended rifted continental margin [6,9,11], but presently it occurs as a continental allochthon [21]. Furthermore, a small oceanic basin is assumed to disappear during the continental convergence [6]. In this study, we report for the first time the occurrence of coesite inclusions in garnet of one jadeite-bearing orthogneiss from the Sesia Zone in the Western Alps. Laser Raman spectra clearly show the diagnostic peaks of coesite, providing unambiguous evidence that part of the crustal rocks in the Sesia Zone experienced UHP metamorphism at mantle depths. Combining pseudosection modeling and zirconological results, we show that the target rock experienced peak metamorphic pressures of 2.8–3.3 GPa at ca. 76 Ma. In terms of the present constraints on the P–T conditions and metamorphic age for the UHP metamorphism in the Sesia Zone, we are in a position to decipher not only the tectonic evolution of the Sesia Zone in the Western Alps but also the geodynamic mechanism for deep subduction of the continental crust bordering a small oceanic basin.

## GEOLOGICAL BACKGROUND AND SAMPLE

**Figure 1.**
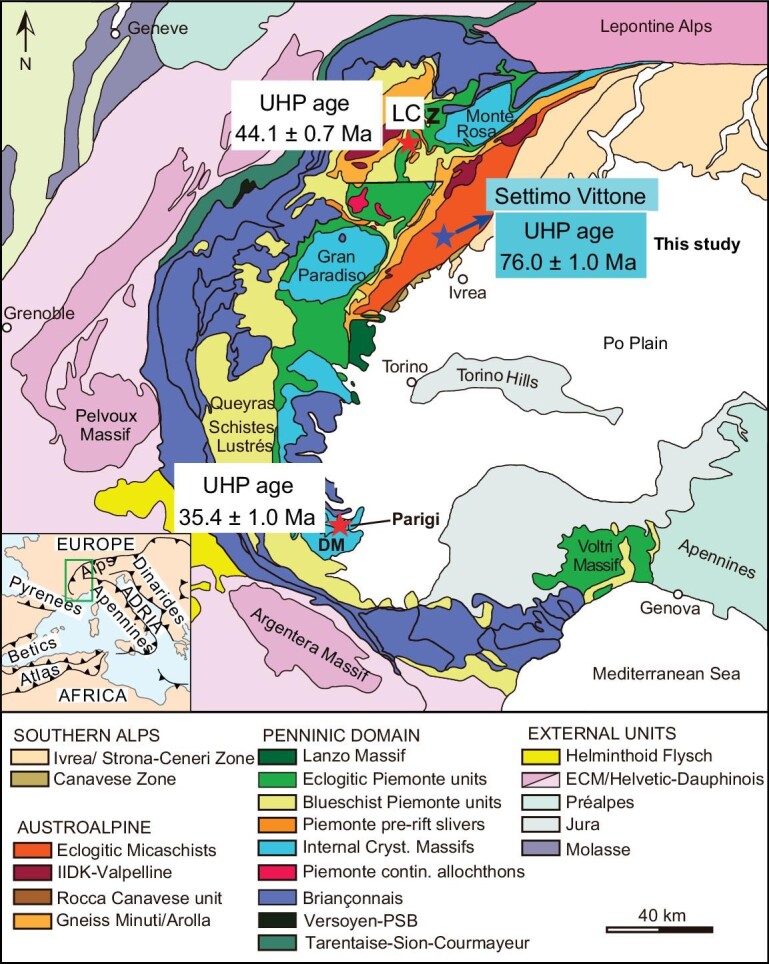
Tectonic map of the Western Alps (adapted from [9]). DM, Dora Maira Massif with the Brossasco-Isasca UHP unit and the locality Parigi (Case Ramello); GP, Gran Paradiso Massif; LC, Lago di Cignana (LC) UHP Unit; MR, Monte Rosa Massif; Z, Zermatt-Saas Zone. The blue star indicates the sample location of Settimo Vittone. The UHP metamorphic ages of Lago di Cignana and Parigi in the Dora-Maira Massif are from [16] and [13], respectively.

**Figure 2.**
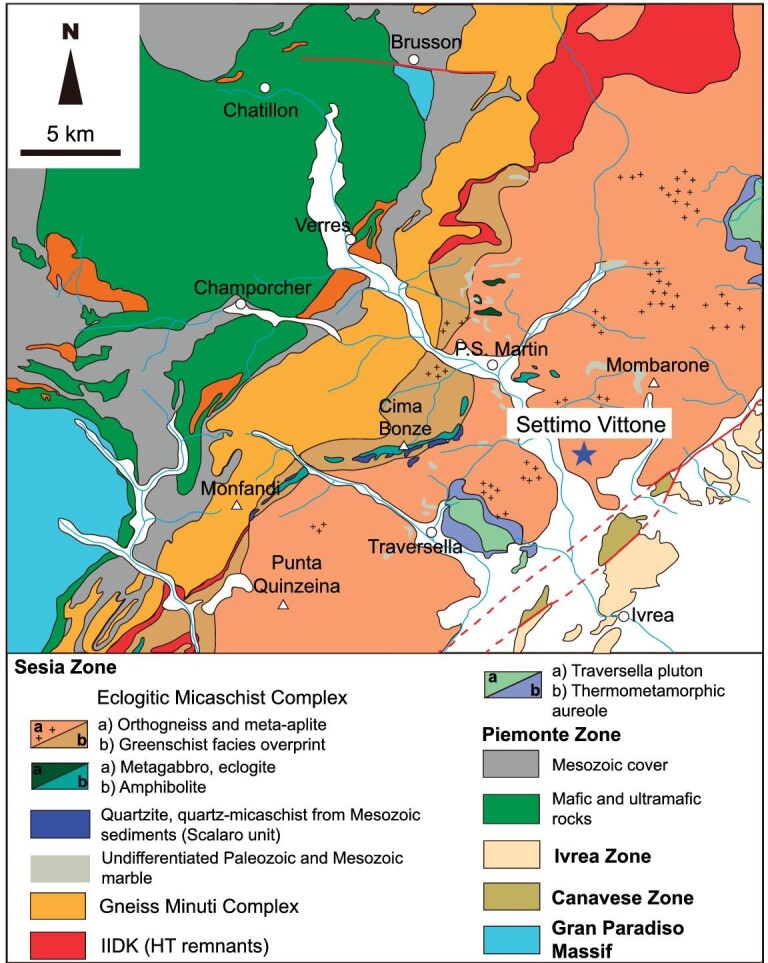
Tectonic map of the central part of the Sesia Zone (adapted from [10,22,25]). The sample locality of Settimo Vittone is shown.

The internal Western Alps includes the Internal Crystalline Massif, Piemont-Liguria Zone and Sesia Zone. The Sesia Zone is located at the eastern part of the Western Alps (Figs 1 and 2). It is bounded by the meta-ophiolites of the Piemont-Liguria Zone to the northwest, the Insubric Line to the southeast and the Lanzo Ultramafic Massif to the southwest. It is a composite unit that mainly consists of the Paleozoic continental basement, which probably belongs to the hyperextended Adriatic margin formed during the opening of the Neo-Tethys Ocean through continental rifting in the Jurassic [9,22]. This basement commonly preserves the Paleozoic imprint metamorphosed at upper amphibolite to granulite facies, which then experienced the eclogite facies HP metamorphism [22]. Although various subdivisions of the Sesia Zone have been suggested, we have adopted here the classical three sub-unit division by [22] that was proposed on the basis of metamorphic grade and main rock types (Figs 1 and 2), i.e. Eclogitic Micaschists Complex (EMC), Gneiss Minuti Complex (GMC) and Second Diorite-Kinzigite Complex (IIDK).

The present study focuses on samples from the EMC, which is a polycyclic basement that mainly consists of paragneiss, with minor amounts of metabasaltic rocks, orthogneiss and impure marble. The basement was intruded by numerous Carboniferous to Permian granitoids and minor gabbros [17,23]. Granitoids are dominant in the northeastern part of the Sesia Zone. Previous studies found that the EMC reached peak metamorphic P–T conditions of 1.5–2.4 GPa and 500–600°C [24,25], which are converted to thermobaric ratios of 250–333°C/GPa. They correspond to low geothermal gradients of 7.5–10°C/km, which belong to Alpine-type eclogite facies metamorphism [26]. The eclogite facies HP metamorphism took place at 80–65 Ma according to the studies of different slices [25,27,28]. Nevertheless, the eclogite facies HP metamorphic slices were exhumed to blueschist facies conditions at 65–60 Ma [20]. A prograde HP metamorphic age of 85.8 ± 1.0 Ma was obtained at P–T conditions of 1.9–2.0 GPa and 540–550°C [25], which are converted to thermobaric ratios of 275–284°C/GPa. They correspond to low geothermal gradients of 8.3–8.5°C/km, which belong to Alpine-type eclogite facies metamorphism [26]. For a slice of the Sesia Zone exposed at the locality of Fondo, south of Quincinetto, a pressure cycling at eclogite facies was proposed with two episodes of HP metamorphism at 80–75 and 70–65 Ma, respectively, with a time lag of less than ca. 20 Myr [25,28].

The sample used in this study was collected from a jadeite-bearing leucocratic orthogneiss quarry (Argentera Quarry; N45°32′25.70″, E7°50′40.27″) at Settimo Vittone, near the village of Quincinetto (Fig. 2). This quarry is an open-pit mine where a ca. 10 meter thick leucocratic orthogneiss layer is thickened by meter-scale parasitic folds. The orthogneiss collected in the nearby (<2 km) outcrops is leucomonzogranitic in composition, with high SiO_2_ content (>73 wt%) and low MgO + FeO + TiO_2_ content (<2.1 wt%), and the reported protolith age is 396 ± 21 to 435 ± 8 Ma [23].

## ANALYTICAL METHODS AND RESULTS

All the analytical methods are presented in Supplementary Data. The present study focuses on orthogneiss sample 13AP41, whose whole-rock major and trace elements, mineral O isotopes, secondary ion mass spectrometry (SIMS) zircon U-Pb age, laser ablation inductively coupled plasma mass spectrometer (LA-ICPMS) zircon U-Pb age and trace elements, as well as mineral major elements, are listed in Tables S1–S6, respectively. The SIMS zircon U-Pb data are listed in Fig. S1. Mineral abbreviations are after [29].

### Petrographic observation and whole-rock geochemistry

**Figure 3.**
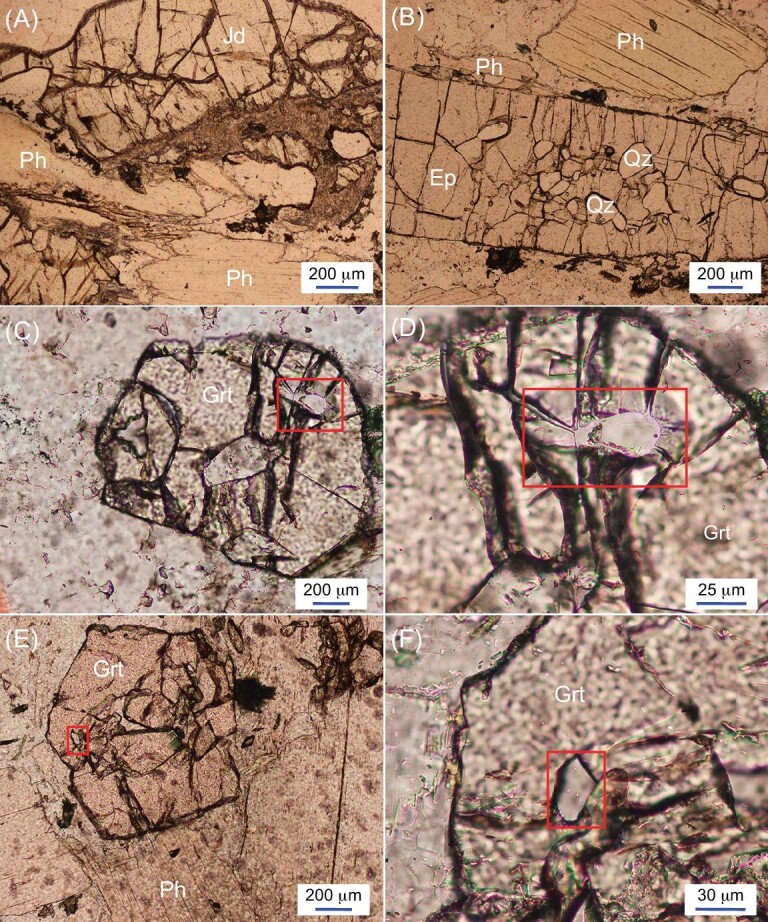
Microphotographs of orthogneiss 13AP41. All images were taken under plane polarized light. The garnet with coesite inclusions is shown in panels (C) and (E), and enlarged in panels (D) and (F), respectively. The sample is mainly composed of quartz, jadeite, phengite and epidote, with minor garnet and titanite. The jadeite grains are surrounded by a thin retrogression corona mainly consisting of albite (A). Quartz grains frequently occur as inclusions in (B) epidote and (C–F) garnet, and some of them show radial cracks in the host minerals (C–F). (C and E) Garnet grains are generally euhedral with little resorption. Mineral abbreviations: Jd, jadeite; Ph, phengite; Ep, epidote; Qz, quartz; Grt, garnet.

Sample 13AP41 collected from the Argentera Quarry is a leucocratic orthogneiss, being primarily composed of quartz, jadeite, phengite and epidote, with minor garnet and titanite (Fig. 3). The jadeite grains are usually big at 1–2 cm in length, and they are commonly surrounded by a thin retrogression corona mainly consisting of albite. Quartz grains frequently occur as inclusions in epidote and garnet, and some of them show radial cracks in the host minerals (Fig. 3). Phengite commonly occurs in the matrix with a narrow retrogression rim. Garnet grains are generally euhedral with negligible resorption.

This sample shows a leucogranitic composition with high SiO_2_ (72.21 wt%) and low MgO + FeO + TiO_2_ content (Table S1), similar to that reported by [23]. It has a high content of large ion lithophile elements (LILEs) such as Ba (805 ppm) and Sr (174 ppm) as well as light rare earth elements (LREEs) such as La (37.1 ppm) and Ce (76.3 ppm), but a low content of high field strength elements (HFSEs) such as Nb (8.2 ppm) and Ta (0.4 ppm) and heavy REEs (HREEs) such as Yb (3.05 ppm) and Lu (0.43 ppm).

### Coesite identification by laser Raman spectroscopy

**Figure 4.**
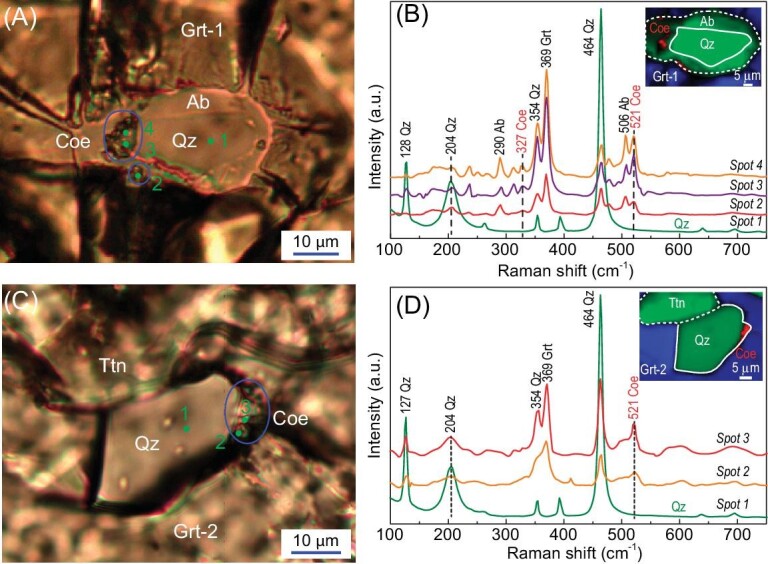
Coesite inclusions in garnet from orthogneiss 13AP41. (A) Coesite and quartz inclusions (below surface) in garnet (Grt-1), corresponding to the microphotographs shown in Fig. S1c–d. (B) Representative laser Raman spectra of coesite shown in panel (A). The inset shows a Raman map indicating the spatial occurrence of coesite inclusions (red color). (C) Coesite and quartz inclusions (below surface) in garnet (Grt-2), corresponding to the microphotographs shown in Fig. S1e–f. (D) Representative laser Raman spectra of coesite shown in panel (C). The inset shows a Raman map indicating the spatial occurrence of coesite inclusion (red color) at the grain boundary of quartz. In addition to Raman peaks of coesite, those of intimately intergrown quartz, albite and garnet occur in the respective patterns (B and C). Mineral abbreviations: Coe, coesite; Qz, quartz; Ttn, titanite; Ab, albite.

Coesite relics were found as inclusions in two garnet grains, Grt-1 and Grt-2 (Fig. 4). Two coesite relics in Grt-1 occur below the surface of the thin section. One large relic occurs along the boundary of quartz and albite, and the small one occurs between albite and garnet (Fig. 4A). There are radial cracks around the coesite + quartz + albite composite inclusion. In Raman spectra, the coesite relics show a significant peak at 521 cm^−1^, with peaks of quartz (464 cm^−1^, 354 cm^−1^, 204 cm^−1^ and 128 cm^−1^), garnet (369 cm^−1^ and 237 cm^−1^) and albite (506 cm^−1^). For comparison, the analysis of solely quartz in coexistence with coesite during the same session lacks the peak of 521 cm^−1^ that is characteristic of coesite (Fig. 4B). The Raman mapping shows the spatial occurrence of the coesite relics, and both relics show a maximum length of 10–15 μm (Fig. 4B).

A further coesite relic was observed inside Grt-2, close to the boundary between quartz and garnet (Fig. 4C). This relic occurs at least ∼5 μm below the surface of the thin section, and shows a strong peak at 521 cm^−1^ along with peaks of quartz and garnet. For comparison, the analysis of solely quartz in coexistence with coesite does not show the peak at 521 cm^−1^. Notably, all of the coesite relics in Grt-1 and Grt-2 exhibit a strong garnet peak at 369 cm^−1^ and a quartz peak at 464 cm^−1^, indicating their subsurface occurrence within garnet. Raman mapping shows that this coesite relic in Grt-2 exhibits a length of ∼10 μm (Fig. 4D).

### Zircon U-Pb ages and trace elements

**Figure 5.**
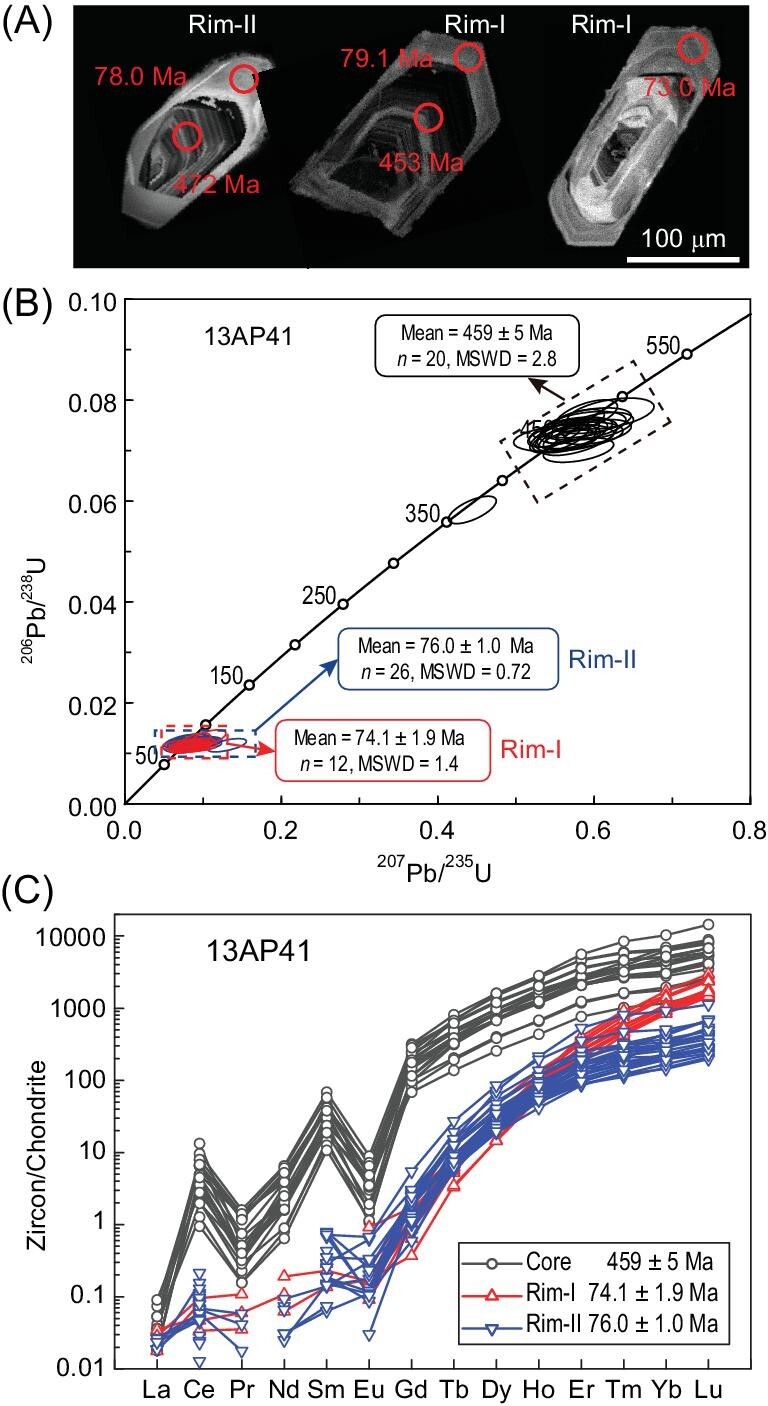
(A) Cathodoluminescence (CL) images, (B) U-Pb isotope concordia diagram and (C) chondrite normalized REE patterns of zircons in orthogneiss 13AP41. Rim-I and Rim-II denote the metamorphic domains with steep HREE patterns and flattened HREE patterns, respectively. Note that many LREE contents of Rim-I and Rim-II domains are below the detection limit and are not plotted. The chondrite values are after [64].

Zircon grains in sample 13AP41 show a clear core-rim structure (Fig. 5). Two zircon mounts were made, one for the SIMS analysis and the other for analysis by LA-ICPMS. The two approaches give consistent results (Figs 5 and S1). The SIMS analysis of zircon cores and rims yields concordant U-Pb ages of 438 ± 6 to 480 ± 7 Ma, and 75.1 ± 1.5 to 78.0 ± 1.4 Ma, respectively, and discordant ones show a ^206^Pb/^238^U age range from 98 to 406 Ma (Fig. S1). Notably, the U-Pb ages obtained by LA-ICPMS can be directly linked to trace element patterns measured at the same spot. Thus, in the following, only the LA-ICPMS U-Pb ages are considered further.

**Figure 6.**
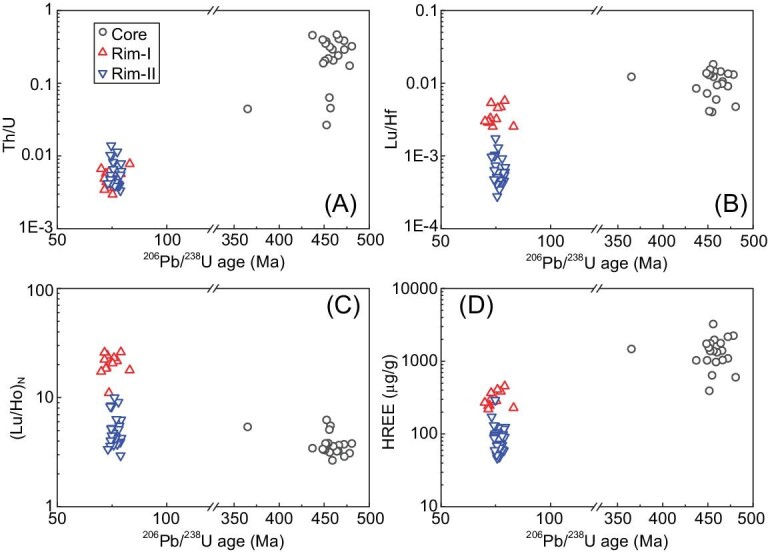
The correlations between zircon trace elements and U-Pb ages for orthogneiss 13AP41. (A) Zircon Th/U ratios, (B) Lu/Hf ratios, (C) (Lu/Ho)_N_ and (D) HREE content, vs. ^206^Pb/^238^U ages. All of the data are analyzed by LA-ICPMS.

The relict cores exhibit oscillatory zoning, high Th/U ratios (mostly >0.1), high REE content with steep HREE patterns and negative Eu anomalies (Figs 5 and 6), suggesting their crystallization from granitic magma at 459 ± 5 Ma. This age is older than that reported for the orthogneiss from the same quarry of 396 ± 21 to 435 ± 8 Ma, which was analyzed by dissolution of bulk zircon grains and could have easily incorporated parts of the young rim domains [23]. Notably, the granitic magma of this age is rare in the Sesia Zone, whereas the early Permian ones are dominant [17].

The rims exhibit no zoning, low Th/U ratios (mostly <0.1) and low REE content (Figs 5 and 6), suggesting a metamorphic origin. However, the rims can be divided into two groups based on their HREE patterns (Figs 5 and 6). Rim-I domains have a weighted mean U-Pb age of 74.1 ± 1.9 Ma (MSWD = 1.4, *n* = 12), and show steep HREE patterns with high (Lu/Ho)_N_ ratios of 11–26, and high HREE content and Lu/Hf ratios. Rim-II domains have a weighted mean U-Pb age of 76.0 ± 1.0 Ma (MSWD = 0.7, *n* = 26), and show flattened HREE patterns with low (Lu/Ho)_N_ ratios of 2.9–10, and low HREE content and Lu/Hf ratios.

### Phase equilibrium modeling

Phase equilibrium modeling was conducted in the MnO–Na_2_O–CaO–K_2_O–FeO–MgO–Al_2_O_3_–SiO_2_–H_2_O–TiO_2_–Fe_2_O_3_ (MnNCKFMASHTO) system. The modeling was performed using THERMOCALC version 3.47 [30] with the internally consistent thermodynamic data set ds62 ([31]; updated in February, 2012). The activity-composition relations for solid-solution phases are from the following sources: garnet [32]; muscovite and silicate melt [33]; plagioclase [34]; epidote [31] and clinopyroxene [35]. Pure phases include quartz, coesite, albite, rutile, lawsonite, titanite and H_2_O. Calculated P-T phase diagrams are contoured for mineral compositions using TCInvestigator [36].

Whole-rock composition was used for the modeling, and the Fe^3+^/ΣFe ratio measured by titration (0.46) was adopted. H_2_O was set to be 4.8 mol.%, comparable to the loss on ignition (LOI) content, which represents the maximum water content in the rock. The modeling yields a stable mineral assemblage of coesite, garnet, phengite, clinopyroxene/jadeite, lawsonite, epidote and rutile under the UHP conditions. The presence of garnet, jadeite, coesite and epidote in the thin section (Fig. 3) is consistent with the results of P–T pseudosection modeling. However, lawsonite was not identified in the thin section since it would easily break down during retrogression to form albite, epidote and quartz. Rutile was not found in the sample due to likely complete replacement by titanite during retrogression. Notably, although titanite can occur at UHP conditions in Ca-rich calcsilicate rocks [37], it is only stable at P–T conditions up to ca. 1.1 GPa at 500^o^C in the metagranitic system [38]. However, it can be preserved metastably, as some metagranites in the Western Alps that have experienced UHP metamorphism still contain magmatic relicts and preserve the original magmatic fabric without deformation [39,40].

**Figure 7.**
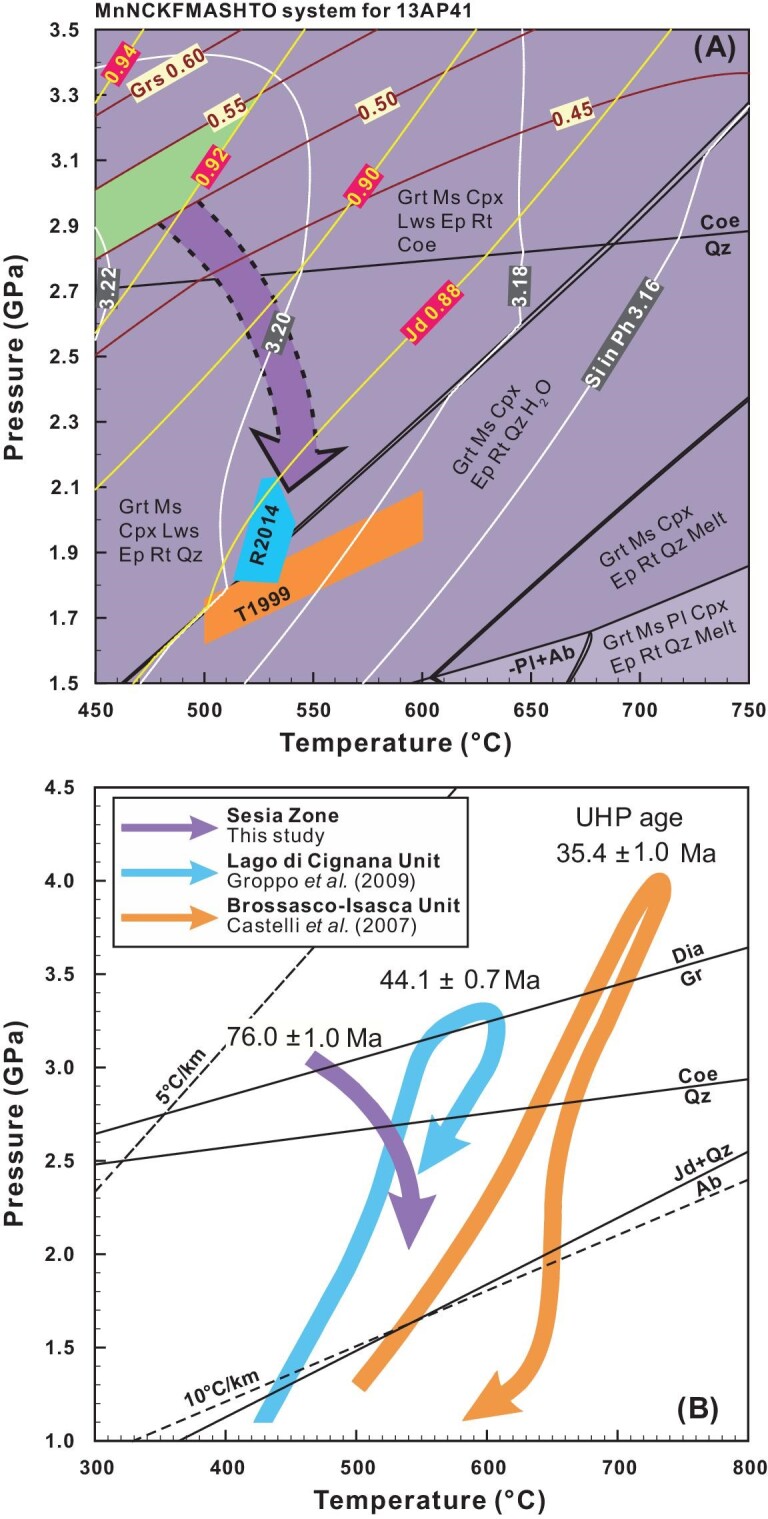
(A) Phase equilibrium modeling results of orthogneiss 13AP41. Whole-rock composition and Fe^3+^/Fe_total_ ratio was used for the calculation (Table S1). Considering the compositions of garnet (Grs: 50%–55%) and jadeite (Jd: >92%) (Table S4), the peak P–T conditions are constrained to be 2.8–3.3 GPa and 450–520°C. The HP metamorphic conditions from [24] and [25] are denoted as T1999 and R2014, respectively. (B) The P–T paths for the other UHP units, i.e. UHP rocks from the Lago di Cignana Unit (oceanic origin), from the Piemont-Liguria Zone and Brossasco-Isasca Unit (continental origin) and from the Dora-Maira Massif are shown for comparison. The P–T path and UHP metamorphic age of the Lago di Cignana Unit are from [65] and [16], respectively, and the later finding of diamond was considered [15]; those for the Brossasco-Isasca Unit are from [66] and [13], respectively.

In order to constrain the peak metamorphic P–T conditions, the chemical compositions of garnet, jadeite and phengite were analyzed. Garnet grains from sample 13AP41 exhibit a grossular component of 43.6–55.2%, jadeites show a jadeite component of 91.6–95.7%, and phengites have an Si pfu (per formula unit, based on O = 11) of 3.22–3.30 (Table S6). Notably, the phengite grains generally contain a core with higher Si pfu content than the rim, suggesting that the core domain formed at peak conditions. Grt-I and Grt-II show irregular major element zonations, and the domains containing coesite have higher grossular content, which likely grew at peak metamorphic conditions. Grossular isopleths of garnet are distributed in near-parallel fashion with shallow positive slopes, and the grossular content increases regularly as pressure increases, making them suitable for the P-T constraint. The isopleths of jadeite component exhibit steep slopes and decrease with increasing temperatures (Fig. 7A). The isopleths of grossular component in garnet, and jadeite component in clinopyroxene, constrain peak metamorphic P–T conditions at ca. 2.8–3.3 GPa and 450–520^o^C (Fig. 7A). The constrained P-T conditions are consistent with results of the measured Si-isopleths of phengite and are located in the stability field of coesite, consistent with the occurrence of coesite inclusions in garnet.

Due to the low Mg content of the studied rock and the lack of Fe-Mg solid solutions in many minerals like garnet and clinopyroxene, the uncertainties of P–T estimates by phase equilibrium modeling in this case can be large, especially for the temperature estimate (ΔT∼ ± 50^o^C, ΔP∼ ± 0.2 kbar). Furthermore, the predicted Si in phengite values are 3.20–3.22, generally consistent with the measured phengite composition (Si_pfu_: 3.20–3.30). It should be noted that since the relevant Si isopleths of phengite, calculated from the whole-rock composition of the rock that was used for modeling, are not suitable for a precise estimation of equilibrium pressures and temperatures, the composition of phengite was only utilized to check consistency with the P–T conditions derived from the X_Grs_ and X_Jd_ data. In addition, Si in phengite is sensitive to the effective whole-rock composition used, thus the analytical errors of phengite composition (±0.5% relative, corresponding to the uncertainty of Si pfu of ±0.01) might lead to a large uncertainty in the P–T constraint. Therefore, the phengite composition was only utilized to check the rationality of the thermodynamic modeling, rather than to determine the precise P–T conditions. The estimated temperature is generally consistent with those obtained by O isotope fractionations between quartz, jadeite and muscovite, i.e. 420–450^o^C (Table S2), considering the thermometric uncertainty, typically ±30–50°C, induced by the uncertainties in O isotope analysis and O isotope fractionation factors between minerals [41].

## THE FIRST EVIDENCE FOR UHP METAMORPHISM IN THE SESIA ZONE

The Raman spectra at 521 cm^−1^ indicate the presence of coesite in the jadeite-bearing orthogneiss 13AP41, providing the first evidence for deep subduction of the continental crust in the Sesia Zone. These data are independently confirmed by phase equilibrium modeling using garnet and jadeite compositions. Notably, the Raman peaks have been checked carefully with different focus depths in the thin section using various laser powers. All of the results give a clear peak at ca. 521 cm^−1^. Although jadeite can show a weak peak at ca. 521 cm^−1^, its main peaks are at ca. 698 cm^−1^ and to a lesser extent, 431 cm^−1^ and 373 cm^−1^ [42]. Thus, if a relict jadeite could occur in Grt-1, the related most significant peak would be expected to occur at 698 cm^−1^. However, such intense peaks at 698 cm^−1^ and 431 cm^−1^ were never found in any analytical results (Fig. 4). Therefore, the Raman peak at ca. 521 cm^−1^ of the inclusion in Grt-1 cannot be caused by the presence of relict jadeite, but instead indicates the presence of coesite. Furthermore, the inclusion in Grt-2 is not associated with any albite or jadeite, thus the presence of 521 cm^−1^ in this grain must reflect the presence of coesite. Combined with the above results, this provides unambiguous evidence for the presence of coesite in the sample.

Phase equilibrium modeling at the isopleths of the grossular component in garnet, and jadeite component in clinopyroxene, places tight constraints on peak metamorphic P–T conditions at ca. 2.8–3.3 GPa and 450–520^o^C (Fig. 7A), corresponding to thermobaric ratios of 158–161°C/GPa. Therefore, the target orthogneiss was subducted to mantle depths of >80 km, experiencing Alpine-type eclogite facies UHP metamorphism at low geothermal gradients of 4.7–4.8°C/km [26]. Nevertheless, the majority of UHP metamorphic rocks were retrogressed into HP ones. As documented in the present study, the albite surrounding quartz in Grt-1 is associated with radial cracks, but the radial cracks are also filled by albite (Fig. 4A). Although we cannot exclude the possibility that the former feature is caused by the retrograde reaction of jadeite + quartz → albite, it can be readily explained by transformation of coesite to quartz to form the radial cracks, into which Na-Al-rich HP fluids would infiltrate to form the albite. This explanation is favorable in view of the occurrence of albite in the radial cracks and the absence of primary jadeite in close proximity to the radial cracks.

The presence of coesite in the sample and the results of P–T pseudosection modeling mean that the three main tectonic units in the Western Alps, i.e. the Sesia Zone (this study), the Piemont-Liguria Zone [14,15,43] and the southern part of the Dora-Maira Massif [1,44], contain crustal rocks that experienced UHP metamorphism. Notably, the coesite relics within garnet of this study occur at grain boundaries of quartz-albite, albite-garnet or quartz-garnet (Fig. 4). This feature is not very common. Previously reported coesite inclusions often occur in the center of polycrystalline quartz grains that are in turn enclosed in eclogite-facies minerals [1,2,14,43]. However, as shown in an example from the Dora-Maira whiteschist, coesite relics are also observed directly at the SiO_2_-garnet boundary (Fig. S2). Coesite was also reported as an intergranular phase along grain boundaries in the UHP rocks from the Dabie-Sulu UHP metamorphic belt [45,46]. The preservation of such coesite grains is probably due to the lack of water during retrogression that would prohibit the complete conversion of coesite to quartz [45,46].

Zircon Rim-I domains show low Th/U ratios (<0.01), steep HREE patterns with high HREE content, and high Lu/Hf and (Lu/Ho)_N_ ratios (Figs 5 and 6), indicating their growth during prograde metamorphism when garnet proportions were still low [47]. Retrogression during exhumation may also lead to the low modal content of garnet. However, garnet grains are generally euhedral with negligible resorption in this sample (Fig. 3), excluding the release of REEs during the retrogression. In contrast, Rim-II domains show flattened HREE patterns and low Lu/Hf and (Lu/Ho)_N_ ratios (Figs 5 and 6), indicating their possible growth during UHP metamorphism when garnet exerted a strong effect on the HREE partition [47]. Although no coesite inclusions were found in metamorphic zircon domains after extensive searches in a large number of grains, the indistinguishable U-Pb ages of Rim-I and Rim-II domains provide a robust constraint on the timing of UHP metamorphism between 74 and 76 Ma, most probably at 76.0 ± 1.0 Ma as indicated by Rim-II domains.

This metamorphic age of 76.0 ± 1.0 Ma is well bracketed by the previously reported two groups of HP eclogite facies metamorphic ages, 80–75 Ma and 70–65 Ma, respectively, in the Sesia Zone [25,28]. However, it significantly predates the UHP age of 44.1 ± 0.7 Ma for the Zermatt-Sass metasediment of the Piemont-Liguria unit [16] and ca. 35.4 ± 1.0 Ma for the whiteschist and granitic gneiss in the Dora-Maira Massif [13,48–50]. It appears that there is a considerable difference in the peak UHP metamorphic ages from the Adriatic margin through the Zermatt-Sass ophiolite to the European continental margin. This difference suggests a polycyclic feature for subduction and exhumation of the oceanic and continental crust during the continental convergence in the Western Alps [9,16].

Notably, the metamorphic rocks have experienced different tectonic histories in different parts of the EMC unit of the Sesia Zone. For example, with the method of *in situ* laser Ar/Ar dating, Halama *et al.* (2014) found that the phengite cores in weakly deformed gneisses from the EMC unit would crystallize at 88–82 Ma, whereas the phengite rims would crystallize at 77–74 Ma [51]. The older Ar/Ar age was consistent with the Rb-Sr isochron age of 85 ± 1 Ma [52] and U/Th-Pb allanite age of 85.8 ± 1.7 to 84.9 ± 2.1 Ma for the micaschist from the Druer slice [25]. Interestingly, Rubatto *et al.* (2011) found that the micaschists from the EMC unit experienced two episodes of HP metamorphism at 79–75 Ma and 70–65 Ma, respectively, arguing that the rock unit experienced two subduction-exhumation cycles in an interval of <20 Myr [15]. By combining the structural, petrological and geochronological observations, furthermore, Regis *et al.* (2014) recognized two independent km-sized tectono-metamorphic slices that experienced different P–T-t evolution paths between 85 and 75 Ma [25].

In comparison, Vho *et al.* (2020) found an age of 67.4 ± 1.9 Ma for zircon rims in the micaschist from Monte Mucrone of the EMC unit, whereas the allanite in metagabbro of the Ivozio Complex gave two metamorphic ages of 62.9 ± 4.2 Ma and 55.3 ± 7.3 Ma, respectively [17]. The presence of zircon overgrowth only in the central area of the EMC unit was attributed to local factors like multiple fluid pulses at HP metamorphic conditions and/or slightly higher temperatures reached in this area during the HP metamorphism [17]. In addition, they compiled the published metamorphic ages of the whole EMC unit of the Sesia Zone, and found different P–T-t paths experienced by HP rocks from different parts. Despite the complexity of metamorphic history experienced by the EMC unit, the previous constrained HP metamorphic ages cluster at 80–65 Ma [11,17,25,28]. The present study gives the UHP metamorphic age of 76.0 ± 1.0 Ma, falling within the proposed metamorphic age range of the HP rocks from the EMC unit [25,28], though the peak metamorphic pressures experienced by the rocks are different.

**Figure 8.**
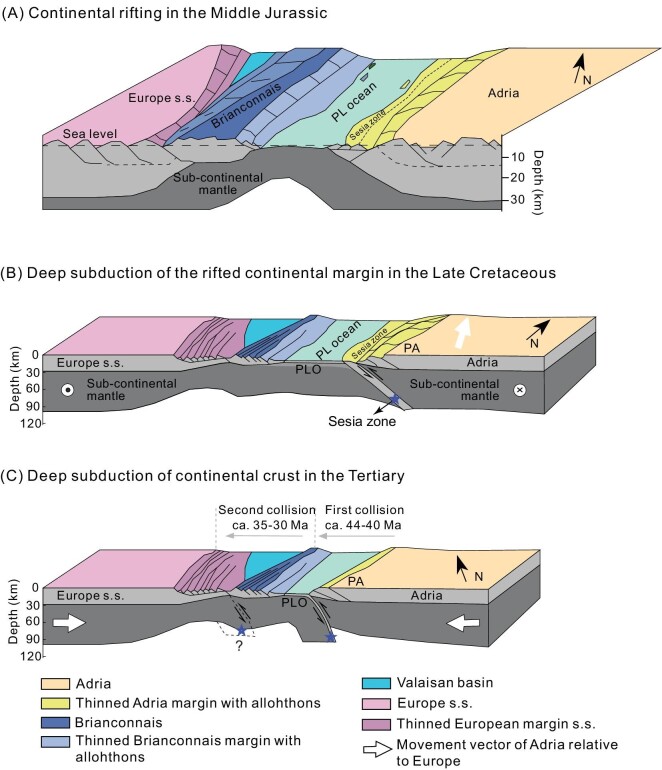
Schematic cartoon for the tectonic evolution of the Sesia Zone and the Western Alps from Jurassic rifting to Tertiary orogeny (adapted from [9]). (A) A simplified Jurassic paleogeography at the onset of mantle peridotite exhumation in the Valais and Piemont-Liguria oceanic basins. The Sesia Zone is a hyperextended continental margin of the Adria plate. (B) During the Late Cretaceous, the oblique subduction of continental crust in the Sesia Zone occurred, resulting in UHP metamorphism at mantle depths of >80 km and forming the proto-Alps terrane. (C) The present Western Alps orogen was formed by the continental collision of the proto-Alps terrane with the Briançonnais terrane and European plate in the Tertiary. The blue stars denote the UHP metamorphic rocks subducted to mantle depths. Abbreviations: PLO, Piemont-Liguria Ocean; PA, proto-Alps.

The exhumation of HP to UHP metamorphic slices in collisional orogens would generally occur in two stages [7]. The first is the slice thrust at lower geothermal gradients, and the second is the domical uplift at higher geothermal gradients. The occurrence of eclogite facies HP to UHP metamorphic rocks in the Sesia Zone indicates their exhumation mainly in the first stage. In doing so, a small change in the relative direction of plate motion and along-strike variations in the convergent plate margin geometry may have great effects on the force balance acting on colliding blocks. Such a change may lead to episodic exhumation of subducted crustal slices along the same continental subduction channel [53], which can account for the two episodes of HP metamorphism in the Sesia Zone [25,28]. Babist *et al.* (2006) documented that early exhumation of the EMC to blueschist facies conditions and juxtaposition to the IIDK occurred at ca. 65–60 Ma [20]. A tectonic process like shearing, between the EMC and IIDK units, could dominate the early exhumation of UHP slices in the Sesia Zone [20]. In any case, the early exhumation of the EMC well predates the closure of the Piemont-Liguria Ocean in the Paleogene. After ca. 60 Ma, the Africa plate would move toward NNW with respect to the Europe plate [19]. Considering the P–T paths of HP and UHP metamorphic units in the main parts of the Western Alps (Fig. 7), the continental convergence leading to the closure of the Piemont-Liguria Ocean and Valais Ocean would proceed sequentially, eventually resulting in continental deep subduction as recorded by the UHP metamorphic slices (Fig. 8).

## IMPLICATIONS FOR SUBDUCTION DYNAMICS IN THE SESIA ZONE

The present study shows that the continental rock in the Sesia Zone was subducted to mantle depths of >80 km for UHP metamorphism at 76.0 ± 1.0 Ma, which is well before the closure of the Piemont-Liguria Ocean [20]. This process cannot be explained by the gravitational pull of the subducting Piemont-Liguria oceanic lithosphere that occurred since ca. 160 Ma [11,18], because a narrow oceanic basin has been proposed to separate the Sesia Zone from the Adria plate [11,19]. Notably, the Canavese Zone in the westernmost part of the Western Alps was hypothesized as a narrow oceanic basin before the formation of the Sesia Zone [54–56]. This is mainly inferred from the observation that this zone exposes different parts of the distal Adriatic margin, where rift-related thinning led to the local exhumation of mantle peridotite to the seafloor level. In some localities, serpentinized peridotite slivers occur directly in contact with radiolarian chert, indicating that the fossil oceanic basin would have formed since the Jurassic [57]. The geochemical composition of serpentinites is also consistent with an abyssal origin in a hyperextended rifted margin rather than a mid-ocean ridge setting [55]. Therefore, the Canavese Zone may represent the missing oceanic basin prior to the formation of the Sesia Zone, and this basin was narrow and embryonic.

It is generally assumed that deep subduction of the continental lithosphere is spontaneous due to gravitational sinking of the subducting oceanic lithosphere because of its high density at mantle depths of >80 km. This assumption is only valid for the continental crust bordering large oceanic basins, where the subduction of oceanic slabs to deeper mantle depths of >120 km does spontaneously pull the continental lithosphere to shallower mantle depths of 80–120 km. With respect to the continental deep subduction in the Sesia Zone, however, there would only be the narrow oceanic basin represented possibly by the Canavese Zone between the Sesia Zone and Adria plate. Furthermore, no HP to UHP metamorphic rocks in the Sesia Zone were found to have the protolith of oceanic crust [56], otherwise difficulty would be encountered in exhuming the dense, eclogitized oceanic crust [12,58]. Instead, there may be the absence of a large oceanic basin at that time. In addition, the Sesia Zone was proposed to represent a hyperextended passive continental margin of the Adria plate [6,9]. Moreover, the oceanic basin between the Sesia Zone and Adriatic margin were hypothesized to be embryonic and small [11,56]. This is supported by the lack of ophiolites and the complete absence of syn-subduction magmatism in the Sesia Zone and the entire Western Alps [59]. Therefore, subduction of the small oceanic slab cannot provide sufficient force to pull the Sesia continental crust to the mantle depths of ca. 80–120 km [6,60]. In this regard, the slab pull as envisaged by Rosenbaum and Lister (2005) would not be a predominant driving force for the continental deep subduction in the Sesia Zone [19].

A longstanding problem in the tectonic evolution of the Western Alps is the geodynamic mechanism of continental subduction and collisional processes forming the HP and UHP metamorphic units, including the Dora-Maira Massif, the Sesia Zone and the disappeared Piemont-Liguria Ocean. The controversy centers on whether the traditional Benioff-type subduction can apply to the Western Alps, because increasing evidence shows that the tectonic evolution of the Western Alps would proceed from convergence through collision to subduction of hyperextended passive continental margins (Fig. 8), with the disappearance of a small oceanic basin between the convergent continental blocks. This is in stark contrast to Benioff-type subduction, where wide oceanic basins are closed during the subduction of oceanic slabs [6,9,56,60,61]. As argued above, the tectonic and petrological evidence shows that the Sesia Zone probably represents a hyperextended passive continental margin of the Adria plate [6,9]. Due to the lack of oceanic slab pull for continental deep subduction, the other force is required to drive continental deep subduction in this zone.

Notably, paleogeographic and plate kinematic studies suggest that the Sesia Zone was oriented at a high angle to the relative motion direction between African and European plates during the Late Cretaceous, when the Africa plate moved toward NE with respect to the Europe plate, and the oblique subduction occurred [19,62]. The deep subduction of continental crust in the Sesia Zone also occurred in this stage (Fig. 8). During the initial convergence, deformation was concentrated within the Adriatic continental margin like the Sesia Zone, without involvement of the Piemont-Liguria oceanic slab [9]. In this case, the force leading to the tectonic convergence between the Africa plate and European plates is distal plate push (induced) rather than proximal slab pull (spontaneous). Traditionally, the far-field stress of extension for seafloor spreading was ascribed to the distal slab pull (induced). However, this pull force is only effective after subduction of the oceanic slab to mantle depths of >80 km [8], where the oceanic crust undergoes eclogitization to acquire elevated density [63]. In the present case, for the closure of a small oceanic basin in the Sesia Zone, it is likely that the deep subduction of continental lithosphere to ca. 80–120 km would be induced by the far-field stress of compression from the push of either seafloor spreading to generate the mid-ocean ridge or continental breakup through active rifting [8]. In either case, the deep subduction of continental crust bordering small oceanic basins would be geodynamically driven by distal push rather than proximal pull.

## CONCLUSIONS

The continental crust in the Sesia Zone of the Western Alps underwent UHP metamorphism at 76.0 ± 1.0 Ma and 2.8–3.3 GPa. This is the first evidence for the deep subduction of continental crust to mantle depths of >80 km in the Late Cretaceous, considerably predating the closure of the Piemont-Liguria Ocean and subsequent subduction of the oceanic and continental crust to mantle depths in the Early Tertiary. In this regard, continental deep subduction to mantle depths, corresponding to UHP metamorphic conditions in the stability field of coesite, is possibly more common than previously expected but has often been overlooked in collisional orogens. Nevertheless, the exhumation of HP to UHP metamorphic rocks at the convergent plate margin is associated with differential movements of crustal slices, resulting in different sizes of tectonic nappes in the collisional orogen. As a consequence, the exposed HP to UHP rocks in the Sesia Zone show the polycyclic and multiple episodes of continental subduction and exhumation. The occurrence of UHP metamorphic rocks in the Sesia Zone provides insights into the tectonic evolution of the entire Western Alps and other collisional orogens with respect to the geodynamic mechanism of continental deep subduction.

Because the Sesia Zone was transformed from the closure of a small oceanic basin through deep subduction of a hyperextended passive continental margin in the Late Cretaceous, the continental deep subduction in this setting is proposed to be mainly induced by the distal push of either continental breakup or seafloor spreading. As such, the small oceanic basin was closed during the tectonic convergence between the Adria and Europe plates at that time. This type of continental deep subduction can occur with little contribution from the gravitational pull of the subducting oceanic slab when large oceanic basins are closed. Therefore, the deep subduction of continental crust may be driven by different geodynamic mechanisms. Whereas continental crust bordering large oceanic basins would be spontaneously subducted owing to the oceanic slab pull, that bordering small oceanic basins could be subducted due to the far-field stress of compression induced by either continental breakup or seafloor spreading.

## Supplementary Material

nwad023_Supplemental_File
