# Supplementary material for: First finding of continental deep subduction in the Sesia Zone of the Western Alps and implications for subduction dynamics
Source: Natl Sci Rev. 2023 Jan 20;10(5):nwad023. doi: 10.1093/nsr/nwad023 (PMC10089585; doi:10.1093/nsr/nwad023)
Supplement: nwad023_Supplemental_File [file nwad023_supplemental_file.docx]

**Supplemental Materials**

**1. Analytical methods**

**2. Supplemental Tables: Tables S1 to S6**

Table S1. Whole-rock major and trace elements of orthogneiss 13AP41 in the Sesia Zone

Table S2. Mineral O isotopes in orthogneiss 13AP41

Table S3. SIMS zircon U-Pb ages for orthogneiss 13AP41

Table S4. LA-ICPMS zircon U-Pb ages for orthogneiss 13AP41

Table S5. LA-ICPMS zircon trace elements in orthogneiss 13AP41

Table S6. Major elements in garnet, jadeite, muscovite, albite and epidote from orthogneiss 13AP41

**3. Supplemental Figures: Figures S1 and S2**

Figure S1. Zircon CL images (A) and U-Pb concordia diagram analyzed by SIMS (B) for orthogneiss 13AP41

Figure S2. Coesite and coexisting quartz inclusions in the pyrope garnet of whiteschist from the Dora-Maira Massif

**1. Analytical methods**

**Whole-rock major and trace elements**

Whole rock major and trace element compositions were determined at ALS Chemex, Guangzhou, using a PANalytical Axios X-ray spectrometer. A sample of ~0.9 g was added to 9.0 g of Lithium Borate Flux (1:1 Li_2_B_4_O_7_-LiBO_2_), mixed well and fused in an auto fluxer between 1050-1100 ^o^C. A flat glass disc was then prepared, and analyzed by the X-ray fluorescence spectrometer. Loss on ignition (LOI) is acquired by the weight difference after ignition at 1000 ^o^C. The analytical precision and accuracy for the major elements was estimated to be typically better than ±1%. The FeO content was measured by redox titration using K_2_Cr_2_O_7_ solution following the protocols described by Chen et al. (2019). Fe_2_O_3_ and Fe^3+^/∑Fe were calculated from the total iron of whole rock. The accuracy of FeO contents was estimated by measuring multiple silicate standards, including GSP-2 (granodiorite), GSR-1 (granite), and GSR-14 (granitic gneiss), and all are better than ±10% (Chen et al., 2019).

For trace element analyses, a sample of 0.20 g is added to lithium metaborate flux (0.90 g), mixed well and fused in a furnace at 1000 °C. The resulting melt is then cooled and dissolved in 100 mL of 4% HNO_3_/2% HCl solution. This solution is then analyzed by Perkin Elmer Elan 9000 inductively coupled plasma-mass spectrometer (ICPMS). The national silicate standards GSR-1 (granite) and GSR-14 (granitic gneiss) were analyzed for quality control. The precision and accuracy for most trace elements are estimated to be better than ±10%.

**Mineral O isotopes**

Oxygen isotopes of mineral separates were measured by the laser fluorination method using a 25W MIR-10 CO_2_ laser at the CAS Key Laboratory of Crust-Mantle Materials and Environments in USTC. The instrumental conditions and data acquisition were generally described by Zheng et al. (1998, 2002). O_2_ was transferred to a Delta+ mass spectrometer for ^18^O/^16^O measurement. The data are reported using the standard δ^18^O notation relative to VSMOW. Three reference minerals were used: δ^18^O = +11.1‰ for GBW04409 quartz (Zheng et al., 1998), and δ^18^O = +3.7‰ for in house standard 04BXL07 garnet (Gong et al., 2007). Reproducibility of each standard on a given day was better than ±0.2‰ (2σ) for δ^18^O values.

**Mineral inclusion analysis by laser Raman spectrometer**

The Raman spectra of mineral inclusions were obtained using a HORIBA Jobin-Yvon LabRAM HR Evolution confocal Raman mirco-spectrometer at the CAS Key Laboratory of Crust-Mantle Materials and Environments in University of Science and Technology of China (USTC), Hefei. The instrument was equipped with a 532 nm laser and a confocal optical, Air-cooled CCD detector. The spatial resolution was as ~0.5 μm, with a maximum laser power 100 mW. The pinhole diameter of 25 μm was used, and the corresponding spectral resolution was approximately 1.0 cm^-1^ with a grating of 600 lines per mm. The working conditions for each point were ~1 μm beam diameter, 100 μm slit width, 5s acquisition time and 2× accumulations at the room conditions of ~23 °C and 1 bar. The objective used was 100×, the spectra range was 100 to 3500 cm^−1^. The spectrometer was calibrated using the neon lines and a silicon standard. During the analytical sessions, the monocrystalline silicon standard was analyzed in order to check the precision and accuracy of the Raman data.

The Raman maps for quartz/coesite inclusions within garnet were created in two-dimensional mode from 0.5 μm step size. Two areas of ca. 35×60 μm and 40×50 μm below the surface of thin section were scanned by the laser beam. Raman spectra were processed for band positions and intensities of diagnostic 464 cm^-1^ (quartz), 521 cm^-1^ (coesite), 910 cm^-1^ (garnet), and 609 cm^-1^ (titanite) bands. The software HORIBA Scientific’s LabSpec 6 was used for instrument control and data processing.

**Mineral major element analysis**

The major element contents of rock-forming minerals were analyzed with a JEOL JXA-8100 electron microprobe (EMP) at the Institute of Geology and Geophysics, Chinese Academy of Sciences. The analytical conditions are 15 kV accelerating voltage, 20 nA beam current. The spot diameter is 1 μm for garnet and jadeite, 5 μm for zoisite and phengite/muscovite. The counting time for the minerals is 20 s on the peak and 10 s on the lower and upper background positions. Natural minerals and synthetic oxides were used as standards, and a program based on the ZAF procedure was used for data correction. The analytical uncertainties for most major oxides are better than ±1%.

**Zircon CL images**

Zircon was extracted by standard density and magnetic separation techniques, and then selected by hand picking under a binocular microscope. For U-Pb isotope analyses, zircon separates were mounted in epoxy resin and polished down to expose the grain center. In order to search mineral inclusions (especially coesite) in zircon, two mounts were made, one for SIMS U-Pb analysis, one for LA-ICPMS analysis. The mount was carefully prepared to keep the polished surface flat. The morphology and internal structure of zircon were studied with the binocular microscope and by cathodoluminescence (CL) imaging. The CL images were collected at the CAS Key Laboratory of Crust-Mantle Materials and Environments in University of Science and Technology of China (USTC), Hefei, using a Tescan Mira 3 LMH Field Emission-Scanning Electron Microprobe (FE-SEM). During the CL imaging, the accelerating potential was 20 kV and beam current was 15 nA.

**SIMS zircon U-Pb ages**

Zircon U-Pb isotopes were measured by Cameca IMS 1280 ion microprobe at the State Key Laboratory of Isotope Geochemistry in Guangzhou Institute of Geochemistry, Chinese Academy of Sciences, Guangzhou. The instrumentation and operating conditions followed Li et al. (2009). Pb/U calibration was performed relative to the zircon standard Plešovice (Sláma et al., 2008) following the method of Li et al. (2009). Calculation of U and Th contents for unknowns was based on the observed correlations between ^238^U^16^O_2_, ^232^Th^16^O and ^90^Zr_2_^16^O for zircon standard 91500 (Li et al., 2009). The model crustal Pb isotope composition of Stacey and Kramers (1975) was used for the common Pb correction. In most cases, the common Pb correction was not sensitive to the choice of common Pb composition. Uncertainty of preferred values for the external standard 91500 was propagated to the final results of the samples. U-Pb concordia diagrams and weighted mean calculations were made using Isoplot/Ex_ver3.23 (Ludwig, 2003). Twelve measurements of zircon standard Qinghu during the analytical session yielded a ^206^Pb/^238^U age mean of 160 ± 4 Ma (2 sd), well consistent within error with the value of 159.5 ± 0.2 Ma (2sd) recommended by Li et al. (2013).

**LA-ICPMS zircon U-Pb ages and trace elements**

The laser ablation (LA)-ICPMS analyses of zircon trace elements and U-Pb ages were conducted at the CAS Key Laboratory of Crust-Mantle Materials and Environments in University of Science and Technology of China (USTC), Hefei. An Agilent 7900 Q-ICPMS was connected to a GeoLas2005 193 nm excimer ArF laser-ablation system. Helium was used as the carrier gas, and argon as the make-up gas was mixed with the carrier gas via a T-connector before entering the ICP. Each analysis incorporated a background acquisition of ~20-30 seconds (gas blank) followed by 50 seconds data acquisition from the sample. Analyses of the zircons were conducted with a laser repetition rate of 6 Hz and beam diameter of 24 μm. The analytical procedures follow those described by Liu et al. (2008, 2010). The raw data procession for trace elements and U-Pb isotopes were performed by the EXCEL software *ICPMSDataCal.*

Zircon 91500 was used as external standard for the U-Pb dating, which was analyzed twice every 5 analyses of unknowns. Time-dependent drifts of U-Th-Pb isotopic ratios were corrected using a linear interpolation for every five analyses according to the variations of 91500. Zircon GJ-1 was analyzed as unknown to monitor the data quality. Uncertainty of preferred values for the external standard 91500 was propagated to the ultimate results of the samples. Trace element concentrations were calibrated using ^29^Si as internal calibrant and NIST 610 as external reference material. Based on the replicate analyses of the reference material, the precision and accuracy of the analyses were found to be better than ±10% (2σ) for most trace elements.

**2. Supplemental Tables**

Table S1. Whole-rock major and trace elements in orthogneiss 13AP41 from the Sesia Zone

| Sample | 13AP41 |
| --- | --- |
| SiO_2_ (wt.%) | 72.21 |
| TiO_2_ | 0.18 |
| Al_2_O_3_ | 14.77 |
| Fe_2_O_3_ | 1.08 |
| FeO | 1.13 |
| MnO | 0.03 |
| MgO | 0.47 |
| CaO | 1.86 |
| Na_2_O | 3.23 |
| K_2_O | 2.98 |
| P_2_O_5_ | 0.06 |
| BaO | 0.07 |
| LOI | 1.62 |
| Total | 99.82 |
| Cs (µg/g) | 1.16 |
| Rb | 80.4 |
| Ba | 805 |
| Th | 14.0 |
| U | 1.75 |
| Nb | 8.2 |
| Ta | 0.4 |
| Sr | 174 |
| Zr | 135 |
| Hf | 4.3 |
| Y | 40.9 |
| La | 37.1 |
| Ce | 76.3 |
| Pr | 8.99 |
| Nd | 34.0 |
| Sm | 7.64 |
| Eu | 0.87 |
| Gd | 6.60 |
| Tb | 1.06 |
| Dy | 6.84 |
| Ho | 1.41 |
| Er | 4.01 |
| Tm | 0.53 |
| Yb | 3.05 |
| Lu | 0.43 |

Note: (1) LOI, loss on ignition;

(2) The whole-rock major elements are in wt.%, and the trace elements are in unit of µg/g.

Table S2. Mineral O isotopes in orthogneiss 13AP41

| Sample | Mineral | δ^18^O (‰) | Mineral pair | ∆^18^O (‰) | T (°C) |
| --- | --- | --- | --- | --- | --- |
| 13AP41 | quartz | 11.2 |  |  |  |
|  | jadeite | 7.9 | quartz-jadeite | 3.3 | 421 |
|  | muscovite | 7.7 | quartz-muscovite | 3.5 | 447 |

Note: The O isotope fractionation equations are after Zheng (1993a,b). Jadeite is assumed with Di_10_Jd_90_, garnet with composition of Grs_55_Alm_45_. The uncertainty of the estimated temperature is ca. ±30-50 °C, by considering the errors induced by O isotope analysis and the O isotope fractionation factors between minerals (Zheng et al., 1998). The δ values of O isotope compositions are presented relative to VSMOW.

Table S3. SIMS zircon U-Pb ages for orthogneiss 13AP41

| Spot | Th | U | Th/U | ^206^Pb_c_  (%) | Isotope ratios | | | | | | Apparent ages (Ma) | | | |
| --- | --- | --- | --- | --- | --- | --- | --- | --- | --- | --- | --- | --- | --- | --- |
|  | µg/g | µg/g |  |  | ^207^Pb/^206^Pb | 1σ (%) | ^207^Pb/^235^U | 1σ (%) | ^206^Pb/^238^U | 1σ (%) | ^207^Pb/^235^U | 1σ | ^206^Pb/^238^U | 1σ |
| 1 | 372 | 1468 | 0.25 | 0.01 | 0.05667 | 0.3 | 0.56758 | 1.5 | 0.0726 | 1.5 | 456 | 6 | 452 | 7 |
| 2 | 5.3 | 305 | 0.02 | 0.05 | 0.05600 | 0.6 | 0.48177 | 1.6 | 0.0624 | 1.5 | 399 | 5 | 390 | 6 |
| 3 | 0.4 | 73 | 0.01 | 0.45 | 0.06265 | 4.3 | 0.10417 | 4.7 | 0.0121 | 1.7 | 101 | 4 | 77.3 | 1.3 |
| 4 | 2.2 | 75 | 0.03 | 2.78 | 0.06178 | 14.6 | 0.13023 | 15.0 | 0.0153 | 3.7 | 124 | 18 | 98 | 4 |
| 5 | 43.8 | 1998 | 0.02 | 0.01 | 0.05681 | 0.4 | 0.60501 | 1.5 | 0.0772 | 1.5 | 480 | 6 | 480 | 7 |
| 6 | 0.3 | 63 | 0.00 | 0.80 | 0.04924 | 6.7 | 0.08262 | 6.9 | 0.0122 | 1.8 | 80.6 | 5.4 | 78.0 | 1.4 |
| 7 | 80.0 | 596 | 0.13 | 0.07 | 0.05735 | 0.9 | 0.43172 | 2.5 | 0.0546 | 2.3 | 364 | 8 | 343 | 8 |
| 8 | 9.6 | 373 | 0.03 | 0.07 | 0.05594 | 1.2 | 0.23500 | 9.2 | 0.0305 | 9.2 | 214 | 18 | 193 | 18 |
| 9 | 131 | 374 | 0.35 | 0.04 | 0.05578 | 0.7 | 0.50031 | 1.7 | 0.0651 | 1.5 | 412 | 6 | 406 | 6 |
| 10 | 133 | 360 | 0.37 | 0.14 | 0.06081 | 2.5 | 0.52160 | 4.9 | 0.0622 | 4.3 | 426 | 17 | 389 | 16 |
| 11 | 0.2 | 49 | 0.00 | 4.06 | 0.04741 | 21.8 | 0.07657 | 21.9 | 0.0117 | 2.0 | 74.9 | 15.9 | 75.1 | 1.5 |
| 12 | 3.4 | 476 | 0.01 | 0.22 | 0.04862 | 1.7 | 0.07968 | 2.3 | 0.0119 | 1.5 | 77.8 | 1.7 | 76.2 | 1.2 |
| 13 | 149 | 727 | 0.21 | 0.05 | 0.05595 | 0.6 | 0.47420 | 1.6 | 0.0615 | 1.5 | 394 | 5 | 385 | 6 |
| 14 | 6.8 | 129 | 0.05 | 0.30 | 0.05351 | 2.2 | 0.21667 | 2.9 | 0.0294 | 1.9 | 199 | 5 | 187 | 3 |
| 15 | 0.4 | 90 | 0.00 | 0.20 | 0.05523 | 3.7 | 0.09046 | 4.1 | 0.0119 | 1.7 | 87.9 | 3.4 | 76.1 | 1.3 |
| 16 | 54.1 | 174 | 0.31 | 0.21 | 0.05632 | 1.7 | 0.46164 | 2.3 | 0.0594 | 1.5 | 385 | 7 | 372 | 6 |
| 17 | 3.2 | 252 | 0.01 | 0.08 | 0.05838 | 1.4 | 0.40704 | 3.4 | 0.0506 | 3.1 | 347 | 10 | 318 | 10 |
| 18 | 186 | 492 | 0.38 | 0.04 | 0.05610 | 0.7 | 0.56491 | 2.0 | 0.0730 | 1.9 | 455 | 7 | 454 | 8 |
| 19 | 208 | 3321 | 0.06 | 0.01 | 0.05627 | 0.2 | 0.54536 | 1.5 | 0.0703 | 1.5 | 442 | 5 | 438 | 6 |

Note: ^206^Pb_c_ = (^206^Pb/^204^Pb)_c_*(^204^Pb/^206^Pb)_m_, with (^204^Pb/^206^Pb)_m_ the measured ^204^Pb/^206^Pb ratio corrected for background, and (^206^Pb/^204^Pb)_c_ the common Pb ratio of the analyzed spot, calculated from its U-Pb age.

Table S4. LA-ICPMS zircon U-Pb ages for orthogneiss 13AP41

| Spot | Group | Th | U | Th/U | Isotope ratios | | | | | | Apparent ages (Ma) | | | | Concordance  (%) |
| --- | --- | --- | --- | --- | --- | --- | --- | --- | --- | --- | --- | --- | --- | --- | --- |
|  |  | µg/g | µg/g |  | ^207^Pb/^206^Pb | 1σ | ^207^Pb/^235^U | 1σ | ^206^Pb/^238^U | 1σ | ^207^Pb/^235^U | 1σ | ^206^Pb/^238^U | 1σ |  |
| Zircon mount 1 (for LA-ICPMS analysis) | | | | | | | | | | | | | | | |
| 1 | I | 204 | 1007 | 0.20 | 0.0566 | 0.0017 | 0.5664 | 0.0170 | 0.0724 | 0.0011 | 456 | 11 | 451 | 6 | 99 |
| 2 | III | 0.25 | 75.8 | 0.00 | 0.0635 | 0.0095 | 0.0978 | 0.0106 | 0.0123 | 0.0004 | 94.8 | 10 | 78.8 | 3 | 80 |
| 3 | III | 0.24 | 61.1 | 0.00 | 0.0649 | 0.0116 | 0.0828 | 0.0095 | 0.0120 | 0.0006 | 80.8 | 9 | 76.8 | 4 | 95 |
| 4 | I | 99.1 | 2237 | 0.04 | 0.0554 | 0.0015 | 0.4438 | 0.0128 | 0.0583 | 0.0011 | 373 | 9 | 365 | 7 | 98 |
| 5 | I | 172 | 461 | 0.37 | 0.0580 | 0.0020 | 0.5802 | 0.0209 | 0.0727 | 0.0015 | 465 | 13 | 452 | 9 | 97 |
| 6 | II | 0.86 | 235 | 0.00 | 0.0515 | 0.0063 | 0.0747 | 0.0073 | 0.0114 | 0.0003 | 73.1 | 7 | 73.0 | 2 | 100 |
| 7 | III | 0.42 | 108 | 0.00 | 0.0556 | 0.0082 | 0.0814 | 0.0124 | 0.0119 | 0.0005 | 79.4 | 12 | 76.2 | 3 | 96 |
| 8 | I | 335 | 1066 | 0.31 | 0.0569 | 0.0019 | 0.5729 | 0.0184 | 0.0733 | 0.0012 | 460 | 12 | 456 | 7 | 99 |
| 9 | I | 60.7 | 2278 | 0.03 | 0.0554 | 0.0014 | 0.5569 | 0.0159 | 0.0728 | 0.0013 | 449 | 10 | 453 | 8 | 99 |
| 10 | I | 86.8 | 1914 | 0.05 | 0.0546 | 0.0014 | 0.5510 | 0.0144 | 0.0735 | 0.0011 | 446 | 9 | 457 | 7 | 98 |
| 11 | III | 0.58 | 148 | 0.00 | 0.0496 | 0.0075 | 0.0835 | 0.0089 | 0.0122 | 0.0003 | 81.5 | 8 | 78.1 | 2 | 96 |
| 12 | III | 1.49 | 108 | 0.01 | 0.0611 | 0.0075 | 0.0921 | 0.0086 | 0.0116 | 0.0004 | 89.5 | 8 | 74.6 | 3 | 80 |
| 13 | III | 0.46 | 94.9 | 0.00 | 0.0601 | 0.0081 | 0.0862 | 0.0101 | 0.0118 | 0.0004 | 84.0 | 9 | 75.4 | 3 | 89 |
| 14 | III | 0.59 | 70.7 | 0.01 | 0.0506 | 0.0080 | 0.0880 | 0.0075 | 0.0118 | 0.0004 | 85.6 | 7 | 75.6 | 3 | 87 |
| 15 | III | 0.37 | 88.3 | 0.00 | 0.0550 | 0.0085 | 0.0893 | 0.0102 | 0.0122 | 0.0004 | 86.9 | 10 | 78.0 | 3 | 89 |
| 16 | II | 0.60 | 122 | 0.00 | 0.0578 | 0.0088 | 0.0830 | 0.0090 | 0.0112 | 0.0004 | 80.9 | 8 | 71.6 | 2 | 87 |
| 17 | II | 1.09 | 213 | 0.01 | 0.0477 | 0.0060 | 0.0912 | 0.0087 | 0.0121 | 0.0003 | 88.6 | 8 | 77.3 | 2 | 85 |
| 18 | II | 1.08 | 140 | 0.01 | 0.0583 | 0.0069 | 0.1037 | 0.0092 | 0.0130 | 0.0006 | 100 | 8 | 83.2 | 4 | 80 |
| 19 | III | 0.40 | 72.8 | 0.01 | 0.0910 | 0.0122 | 0.1307 | 0.0101 | 0.0116 | 0.0005 | 125 | 9 | 74.5 | 3 | 33 |
| 20 | II | 1.63 | 288 | 0.01 | 0.0518 | 0.0052 | 0.0860 | 0.0081 | 0.0123 | 0.0007 | 83.7 | 8 | 79.1 | 4 | 94 |
| 21 | II | 0.45 | 153 | 0.00 | 0.0540 | 0.0073 | 0.0803 | 0.0073 | 0.0118 | 0.0004 | 78.4 | 7 | 75.3 | 3 | 96 |
| 22 | II | 0.51 | 148 | 0.00 | 0.0511 | 0.0078 | 0.0754 | 0.0089 | 0.0112 | 0.0004 | 73.8 | 8 | 71.7 | 2 | 97 |
| 23 | II | 0.70 | 122 | 0.01 | 0.0616 | 0.0104 | 0.0835 | 0.0097 | 0.0113 | 0.0004 | 81.4 | 9 | 72.3 | 3 | 87 |
| 24 | III | 0.41 | 83.4 | 0.00 | 0.0628 | 0.0109 | 0.0955 | 0.0115 | 0.0117 | 0.0005 | 92.6 | 11 | 74.9 | 3 | 76 |
| 25 | II | 1.43 | 216 | 0.01 | 0.0516 | 0.0065 | 0.0715 | 0.0075 | 0.0109 | 0.0004 | 70.1 | 7 | 70.1 | 2 | 100 |
| 26 | II | 0.65 | 149 | 0.00 | 0.0586 | 0.0100 | 0.0840 | 0.0126 | 0.0113 | 0.0004 | 81.9 | 12 | 72.6 | 3 | 87 |
| 27 | III | 0.28 | 63.9 | 0.00 | 0.0641 | 0.0175 | 0.0810 | 0.0131 | 0.0120 | 0.0008 | 79.1 | 12 | 76.7 | 5 | 97 |
| 28 | II | 0.61 | 101 | 0.01 | 0.0484 | 0.0091 | 0.0754 | 0.0126 | 0.0115 | 0.0004 | 73.8 | 12 | 73.7 | 3 | 100 |
| 29 | III | 0.29 | 68.7 | 0.00 | 0.0614 | 0.0127 | 0.0820 | 0.0092 | 0.0116 | 0.0005 | 80.1 | 9 | 74.6 | 3 | 93 |

Table S4 (continued)

| Spot | Group | Th | U | Th/U | Isotope ratios | | | | | | Apparent ages (Ma) | | | | Concordance  (%) |
| --- | --- | --- | --- | --- | --- | --- | --- | --- | --- | --- | --- | --- | --- | --- | --- |
|  |  | µg/g | µg/g |  | ^207^Pb/^206^Pb | 1σ | ^207^Pb/^235^U | 1σ | ^206^Pb/^238^U | 1σ | ^207^Pb/^235^U | 1σ | ^206^Pb/^238^U | 1σ |  |
| Zircon mount 2 (for SIMS analysis) | | | | | | | | | | | | | | | |
| 1 | I | 446 | 1555 | 0.29 | 0.0555 | 0.0013 | 0.5815 | 0.0134 | 0.0760 | 0.0009 | 465 | 9 | 472 | 6 | 99 |
| 2 | III | 0.56 | 137 | 0.00 | 0.0567 | 0.0079 | 0.0856 | 0.0091 | 0.0122 | 0.0004 | 83.4 | 9 | 78.0 | 2 | 93 |
| 3 | III | 0.55 | 69.5 | 0.01 | 0.0556 | 0.0085 | 0.0871 | 0.0082 | 0.0118 | 0.0005 | 84.8 | 8 | 75.8 | 3 | 88 |
| 4 | III | 0.14 | 36.0 | 0.00 | 0.0596 | 0.0141 | 0.0821 | 0.0099 | 0.0122 | 0.0006 | 80.1 | 9 | 78.4 | 4 | 98 |
| 5 | III | 0.32 | 80.1 | 0.00 | 0.0478 | 0.0077 | 0.0781 | 0.0097 | 0.0120 | 0.0004 | 76.3 | 9 | 77.1 | 3 | 99 |
| 6 | III | 0.99 | 161 | 0.01 | 0.0530 | 0.0047 | 0.0888 | 0.0074 | 0.0124 | 0.0003 | 86.4 | 7 | 79.4 | 2 | 91 |
| 7 | III | 0.87 | 76.3 | 0.01 | 0.0588 | 0.0096 | 0.0957 | 0.0109 | 0.0121 | 0.0004 | 92.8 | 10 | 77.4 | 3 | 80 |
| 8 | I | 64.9 | 299 | 0.22 | 0.0574 | 0.0023 | 0.5773 | 0.0246 | 0.0731 | 0.0011 | 463 | 16 | 455 | 7 | 98 |
| 9 | III | 0.73 | 72.5 | 0.01 | 0.0497 | 0.0072 | 0.0771 | 0.0075 | 0.0117 | 0.0005 | 75.4 | 7 | 74.9 | 3 | 99 |
| 10 | I | 219 | 1262 | 0.17 | 0.0555 | 0.0013 | 0.5908 | 0.0164 | 0.0770 | 0.0013 | 471 | 10 | 478 | 8 | 99 |
| 11 | III | 1.12 | 109 | 0.01 | 0.0466 | 0.0075 | 0.0838 | 0.0097 | 0.0116 | 0.0004 | 81.7 | 9 | 74.3 | 2 | 90 |
| 12 | I | 157 | 538 | 0.29 | 0.0589 | 0.0020 | 0.6006 | 0.0194 | 0.0738 | 0.0010 | 478 | 12 | 459 | 6 | 96 |
| 13 | III | 1.62 | 385 | 0.00 | 0.0509 | 0.0043 | 0.0798 | 0.0063 | 0.0114 | 0.0002 | 78.0 | 6 | 73.1 | 1 | 93 |
| 14 | III | 0.72 | 109 | 0.01 | 0.0481 | 0.0072 | 0.0796 | 0.0103 | 0.0118 | 0.0004 | 77.8 | 10 | 75.7 | 3 | 97 |
| 15 | III | 0.61 | 128 | 0.00 | 0.0509 | 0.0061 | 0.0773 | 0.0068 | 0.0116 | 0.0003 | 75.6 | 6 | 74.3 | 2 | 98 |
| 16 | I | 263 | 569 | 0.46 | 0.0562 | 0.0019 | 0.5797 | 0.0183 | 0.0746 | 0.0008 | 464 | 12 | 464 | 5 | 100 |
| 17 | I | 169 | 708 | 0.24 | 0.0587 | 0.0021 | 0.6054 | 0.0188 | 0.0749 | 0.0011 | 481 | 12 | 466 | 7 | 97 |
| 18 | II | 1.26 | 223 | 0.01 | 0.0506 | 0.0044 | 0.0792 | 0.0052 | 0.0118 | 0.0003 | 77.4 | 5 | 75.9 | 2 | 98 |
| 19 | III | 0.72 | 130 | 0.01 | 0.0514 | 0.0065 | 0.0932 | 0.0097 | 0.0123 | 0.0003 | 90.4 | 9 | 78.9 | 2 | 85 |
| 20 | I | 178 | 392 | 0.45 | 0.0599 | 0.0018 | 0.5808 | 0.0184 | 0.0702 | 0.0010 | 465 | 12 | 437 | 6 | 94 |
| 21 | I | 104 | 507 | 0.20 | 0.0556 | 0.0014 | 0.5694 | 0.0156 | 0.0740 | 0.0010 | 458 | 10 | 460 | 6 | 99 |
| 22 | I | 115 | 283 | 0.41 | 0.0547 | 0.0023 | 0.5876 | 0.0223 | 0.0750 | 0.0012 | 469 | 14 | 466 | 7 | 99 |
| 23 | I | 231 | 586 | 0.39 | 0.0562 | 0.0016 | 0.5614 | 0.0165 | 0.0721 | 0.0010 | 452 | 11 | 449 | 6 | 99 |
| 24 | III | 0.75 | 94.9 | 0.01 | 0.0424 | 0.0062 | 0.0843 | 0.0087 | 0.0124 | 0.0004 | 82.2 | 8 | 79.2 | 2 | 96 |
| 25 | I | 71.0 | 222 | 0.32 | 0.0582 | 0.0021 | 0.6220 | 0.0228 | 0.0774 | 0.0013 | 491 | 14 | 481 | 8 | 98 |
| 26 | III | 0.74 | 152 | 0.00 | 0.0528 | 0.0063 | 0.0811 | 0.0079 | 0.0117 | 0.0003 | 79.2 | 7 | 74.8 | 2 | 94 |
| 27 | I | 169 | 2677 | 0.06 | 0.0559 | 0.0012 | 0.5666 | 0.0113 | 0.0733 | 0.0008 | 456 | 7 | 456 | 5 | 100 |
| 28 | I | 32.0 | 91.6 | 0.35 | 0.0566 | 0.0028 | 0.5618 | 0.0264 | 0.0726 | 0.0011 | 453 | 17 | 452 | 7 | 100 |

Table S4 (continued)

| Spot | Group | Th | U | Th/U | Isotope ratios | | | | | | Apparent ages (Ma) | | | | Concordance  (%) |
| --- | --- | --- | --- | --- | --- | --- | --- | --- | --- | --- | --- | --- | --- | --- | --- |
|  |  | µg/g | µg/g |  | ^207^Pb/^206^Pb | 1σ | ^207^Pb/^235^U | 1σ | ^206^Pb/^238^U | 1σ | ^207^Pb/^235^U | 1σ | ^206^Pb/^238^U | 1σ |  |
| 29 | I | 145 | 774 | 0.19 | 0.0572 | 0.0014 | 0.5703 | 0.0142 | 0.0721 | 0.0008 | 458 | 9 | 449 | 5 | 98 |
| 30 | I | 116 | 306 | 0.38 | 0.0570 | 0.0019 | 0.5980 | 0.0206 | 0.0760 | 0.0010 | 476 | 13 | 472 | 6 | 99 |

Note: Group I, relict magmatic domains; Group II, metamorphic domains with steep HREE patterns; Group III, metamorphic domains with flattened HREE patterns.

Table S5. LA-ICPMS zircon trace elements in orthogneiss 13AP41

| No. | Group | Al | P | Ti | Y | Hf | Nb | Ta | Th | U | La | Ce | Pr | Nd | Sm | Eu | Gd | Tb | Dy | Ho | Er | Tm | Yb | Lu | T/^o^C |  |
| --- | --- | --- | --- | --- | --- | --- | --- | --- | --- | --- | --- | --- | --- | --- | --- | --- | --- | --- | --- | --- | --- | --- | --- | --- | --- | --- |
| Zircon mount 1 (For LA-ICPMS analysis) | | | | | | | | | | | | | | | | | | | | | | | | | | |
| 1 | I | 3.09 | 1522 | 0.15 | 2802 | 11625 | 5.24 | 2.35 | 204 | 1007 | 0.004 | 2.76 | 0.030 | 0.96 | 4.69 | 0.20 | 39.1 | 18.2 | 246 | 102 | 450 | 93.3 | 849 | 151 | 462 |  |
| 2 | III | 2.68 | 45.7 | 0.19 | 129 | 13137 | 0.75 | 0.30 | 0.25 | 75.8 | bdl | 0.027 | bdl | 0.012 | 0.023 | 0.006 | 0.26 | 0.28 | 6.39 | 3.41 | 16.6 | 3.67 | 33.0 | 5.99 | 474 |  |
| 3 | III | 1.86 | 61.0 | 0.13 | 125 | 13963 | 0.82 | 0.31 | 0.24 | 61.1 | 0.004 | 0.038 | bdl | 0.031 | bdl | 0.002 | 0.26 | 0.23 | 5.40 | 3.29 | 17.7 | 3.95 | 36.4 | 6.49 | 454 |  |
| 4 | I | 25.2 | 1538 | 5.78 | 1999 | 14027 | 10.2 | 5.65 | 99.1 | 2237 | 0.022 | 1.71 | 0.038 | 0.76 | 2.90 | 0.11 | 23.5 | 12.0 | 170 | 70.4 | 344 | 81.3 | 886 | 171 | 697 |  |
| 5 | I | 2.95 | 1218 | 5.88 | 2465 | 9525 | 4.10 | 1.25 | 172 | 461 | 0.008 | 4.04 | 0.12 | 2.50 | 6.37 | 0.42 | 49.3 | 17.4 | 220 | 87.0 | 399 | 86.1 | 764 | 146 | 698 |  |
| 6 | II | 1.72 | 54.8 | 0.21 | 213 | 11188 | 2.19 | 1.04 | 0.86 | 235 | 0.004 | 0.049 | bdl | 0.029 | 0.021 | 0.010 | 0.21 | 0.20 | 6.55 | 5.48 | 48.5 | 17.9 | 240 | 59.7 | 478 |  |
| 7 | III | 1.57 | 40.6 | 0.85 | 137 | 13093 | 1.17 | 0.54 | 0.42 | 108 | bdl | 0.039 | bdl | 0.014 | 0.010 | 0.006 | 0.12 | 0.27 | 6.07 | 3.81 | 22.3 | 6.51 | 76.9 | 17.1 | 557 |  |
| 8 | I | 4.95 | 1345 | 0.79 | 2628 | 10924 | 5.36 | 1.91 | 335 | 1066 | bdl | 4.01 | 0.072 | 1.83 | 5.97 | 0.31 | 47.5 | 18.7 | 246 | 94.0 | 414 | 85.4 | 760 | 133 | 553 |  |
| 9 | I | 11.2 | 1470 | 0.54 | 2214 | 16062 | 4.19 | 3.31 | 60.7 | 2278 | bdl | 0.58 | 0.015 | 0.30 | 1.65 | 0.067 | 19.8 | 11.7 | 181 | 75.5 | 388 | 98.5 | 1065 | 212 | 530 |  |
| 10 | I | 9.23 | 1528 | 2.25 | 2617 | 15340 | 3.72 | 2.70 | 86.8 | 1914 | 0.010 | 0.78 | 0.015 | 0.38 | 2.26 | 0.063 | 23.3 | 14.2 | 212 | 90.6 | 457 | 110 | 1181 | 225 | 623 |  |
| 11 | III | 104 | 14.3 | 1.70 | 107 | 14209 | 1.30 | 0.59 | 0.58 | 148 | bdl | 0.064 | bdl | 0.043 | 0.021 | 0.018 | 0.20 | 0.23 | 4.92 | 2.92 | 15.8 | 3.68 | 35.1 | 6.72 | 603 |  |
| 12 | III | 4.55 | 31.9 | 1.28 | 124 | 12289 | 1.07 | 0.42 | 1.49 | 108 | 0.006 | 0.029 | bdl | bdl | 0.022 | 0.010 | 0.39 | 0.28 | 5.55 | 3.33 | 20.0 | 5.19 | 58.3 | 12.6 | 583 |  |
| 13 | III | 44.4 | 82.5 | 1.20 | 108 | 13897 | 0.85 | 0.42 | 0.46 | 94.9 | 0.006 | 0.031 | bdl | 0.014 | 0.011 | bdl | 0.21 | 0.25 | 5.14 | 2.98 | 14.4 | 3.13 | 28.4 | 5.77 | 580 |  |
| 14 | III | 91.2 | 50.7 | 0.18 | 135 | 13810 | 0.81 | 0.32 | 0.59 | 70.7 | 0.006 | 0.036 | 0.002 | bdl | 0.066 | bdl | 0.22 | 0.28 | 6.09 | 3.71 | 19.8 | 4.21 | 38.2 | 7.06 | 471 |  |
| 15 | III | 3.65 | 6.12 | 0.42 | 143 | 14645 | 0.74 | 0.45 | 0.37 | 88.3 | bdl | 0.042 | 0.006 | bdl | 0.039 | bdl | 0.24 | 0.30 | 5.77 | 3.32 | 19.8 | 5.18 | 55.0 | 13.6 | 516 |  |
| 16 | II | 1.10 | 29.8 | 0.71 | 129 | 12600 | 1.30 | 0.95 | 0.60 | 122 | bdl | 0.021 | 0.003 | bdl | bdl | 0.010 | 0.076 | 0.13 | 3.67 | 3.21 | 28.5 | 10.8 | 142 | 37.4 | 546 |  |
| 17 | II | 5.87 | 27.8 | 0.82 | 269 | 13290 | 2.40 | 0.89 | 1.09 | 213 | bdl | 0.047 | bdl | bdl | bdl | 0.010 | 0.48 | 0.33 | 7.59 | 6.40 | 55.4 | 20.0 | 245 | 62.1 | 555 |  |
| 18 | II | 8.53 | 33.6 | 0.64 | 176 | 14170 | 1.73 | 1.00 | 1.08 | 140 | bdl | 0.063 | bdl | 0.089 | 0.035 | 0.009 | 0.18 | 0.26 | 6.27 | 4.48 | 35.4 | 11.5 | 145 | 36.0 | 539 |  |
| 19 | III | 93.3 | 13.3 | 0.58 | 204 | 14836 | 0.93 | 0.37 | 0.40 | 72.8 | bdl | 0.014 | bdl | bdl | 0.039 | bdl | 0.23 | 0.46 | 8.85 | 5.20 | 27.0 | 5.55 | 49.6 | 9.44 | 534 |  |
| 20 | II | 141 | 35.1 | 1.68 | 270 | 13209 | 3.02 | 1.43 | 1.63 | 288 | bdl | 0.040 | bdl | 0.047 | bdl | 0.010 | 0.26 | 0.30 | 8.07 | 6.46 | 60.0 | 21.8 | 294 | 75.7 | 602 |  |
| 21 | II | 2.36 | 28.5 | 0.21 | 216 | 14365 | 1.98 | 0.80 | 0.45 | 153 | bdl | 0.035 | bdl | bdl | bdl | bdl | 0.31 | 0.23 | 6.91 | 4.98 | 43.7 | 15.0 | 180 | 46.2 | 479 |  |
| 22 | II | 2.18 | 25.9 | 0.37 | 161 | 13732 | 1.98 | 1.30 | 0.51 | 148 | bdl | 0.020 | bdl | bdl | bdl | bdl | bdl | 0.12 | 3.77 | 3.94 | 34.1 | 11.9 | 158 | 39.3 | 509 |  |
| 23 | II | 2.28 | 24.6 | 1.11 | 202 | 14042 | 1.56 | 0.66 | 0.70 | 122 | 0.007 | 0.059 | 0.010 | bdl | bdl | bdl | 0.32 | 0.26 | 7.23 | 4.88 | 37.8 | 12.2 | 155 | 40.5 | 574 |  |
| 24 | III | 1.85 | 7.81 | 0.42 | 218 | 14990 | 1.04 | 0.39 | 0.41 | 83.4 | bdl | 0.015 | bdl | bdl | 0.045 | 0.036 | 0.62 | 0.45 | 9.18 | 5.50 | 29.2 | 6.18 | 55.0 | 11.1 | 516 |  |
| 25 | II | 4.99 | 16.4 | 0.53 | 219 | 14145 | 1.62 | 0.92 | 1.43 | 216 | bdl | 0.070 | bdl | bdl | bdl | 0.011 | 0.19 | 0.28 | 8.24 | 5.47 | 42.5 | 13.3 | 172 | 42.5 | 529 |  |
| 26 | II | 522 | 29.8 | 1.45 | 213 | 13695 | 2.15 | 1.37 | 0.65 | 149 | 0.008 | 0.032 | bdl | bdl | 0.044 | bdl | 0.13 | 0.21 | 6.30 | 5.35 | 42.6 | 14.6 | 189 | 45.1 | 592 |  |
| 27 | III | 4.48 | 17.0 | 0.085 | 116 | 15201 | 0.66 | 0.31 | 0.28 | 63.9 | bdl | 0.008 | bdl | bdl | 0.043 | bdl | 0.17 | 0.21 | 5.88 | 2.79 | 14.7 | 2.84 | 25.5 | 5.29 | 435 |  |
| 28 | II | 1.76 | 26.0 | 0.66 | 319 | 15051 | 2.60 | 1.59 | 0.61 | 101 | bdl | 0.029 | 0.006 | 0.050 | bdl | 0.054 | 0.35 | 0.56 | 11.8 | 7.73 | 53.6 | 15.3 | 169 | 38.3 | 541 |  |
| 29 | III | 8.09 | 19.8 | 0.46 | 114 | 15176 | 0.85 | 0.36 | 0.29 | 68.7 | bdl | 0.056 | bdl | bdl | bdl | 0.012 | 0.22 | 0.23 | 4.98 | 3.02 | 16.0 | 3.58 | 33.0 | 7.03 | 520 |  |

Table S5 (continued)

| No. | Group | Al | P | Ti | Y | Hf | Nb | Ta | Th | U | La | Ce | Pr | Nd | Sm | Eu | Gd | Tb | Dy | Ho | Er | Tm | Yb | Lu | T/^o^C |
| --- | --- | --- | --- | --- | --- | --- | --- | --- | --- | --- | --- | --- | --- | --- | --- | --- | --- | --- | --- | --- | --- | --- | --- | --- | --- |
| Zircon mount 2 (For SIMS analysis) | | | | | | | | | | | | | | | | | | | | | | | | | |
| 1 | I | 5.31 | 2023 | 1.64 | 4577 | 14778 | 8.25 | 3.53 | 446 | 1555 | bdl | 4.88 | 0.038 | 1.61 | 7.66 | 0.19 | 65.1 | 30.2 | 405 | 153 | 765 | 147 | 1065 | 198 | 600 |
| 2 | III | 1.01 | 13.6 | 0.24 | 163 | 16469 | 1.32 | 0.57 | 0.56 | 137 | bdl | 0.048 | bdl | bdl | 0.028 | 0.007 | 0.36 | 0.35 | 8.12 | 3.83 | 22.9 | 4.65 | 35.6 | 6.69 | 485 |
| 3 | III | 0.45 | 30.4 | 0.062 | 255 | 17523 | 1.27 | 0.46 | 0.55 | 69.5 | bdl | 0.015 | bdl | bdl | 0.12 | 0.014 | 0.41 | 0.57 | 11.9 | 6.37 | 35.9 | 7.29 | 52.1 | 10.5 | 420 |
| 4 | III | 0.36 | 30.0 | 0.14 | 175 | 17782 | 0.89 | 0.32 | 0.14 | 36.0 | bdl | 0.029 | bdl | bdl | 0.029 | 0.005 | 0.40 | 0.26 | 7.05 | 4.33 | 28.8 | 6.82 | 52.4 | 10.7 | 460 |
| 5 | III | 1.79 | 37.5 | 0.051 | 128 | 17780 | 1.31 | 0.54 | 0.32 | 80.1 | bdl | 0.034 | bdl | bdl | bdl | bdl | 0.28 | 0.23 | 5.60 | 2.88 | 19.5 | 4.52 | 39.3 | 8.20 | 412 |
| 6 | III | 3.12 | 41.9 | 0.33 | 268 | 17567 | 1.48 | 0.61 | 0.99 | 161 | 0.004 | 0.095 | bdl | bdl | bdl | 0.007 | 0.50 | 0.48 | 12.8 | 6.47 | 41.3 | 7.97 | 63.2 | 12.3 | 502 |
| 7 | III | 1.55 | 18.1 | 0.50 | 174 | 17162 | 1.07 | 0.39 | 0.87 | 76.3 | bdl | 0.054 | bdl | bdl | bdl | 0.007 | 0.54 | 0.27 | 8.14 | 4.35 | 24.2 | 5.26 | 36.4 | 7.32 | 526 |
| 8 | I | 2.55 | 459 | 1.57 | 1170 | 16474 | 2.81 | 1.04 | 64.9 | 299 | bdl | 1.26 | 0.027 | 0.42 | 1.93 | 0.092 | 16.8 | 7.17 | 96.8 | 38.4 | 204 | 41.5 | 331 | 66.2 | 597 |
| 9 | III | 0.26 | 59.0 | 0.94 | 487 | 16342 | 4.34 | 2.02 | 0.73 | 72.5 | bdl | 0.054 | bdl | bdl | bdl | 0.014 | 0.51 | 0.57 | 18.0 | 12.0 | 88.2 | 20.4 | 156 | 28.5 | 563 |
| 10 | I | 0.073 | 1950 | 1.36 | 4564 | 15925 | 5.98 | 2.87 | 219 | 1262 | 0.005 | 2.54 | 0.043 | 0.84 | 5.39 | 0.18 | 51.4 | 26.1 | 377 | 149 | 775 | 153 | 1121 | 208 | 588 |
| 11 | III | 1.84 | 1.22 | 0.41 | 211 | 17750 | 1.30 | 0.51 | 1.12 | 109 | bdl | 0.059 | bdl | bdl | 0.054 | 0.019 | 0.44 | 0.47 | 10.2 | 5.21 | 30.0 | 5.65 | 44.0 | 8.46 | 514 |
| 12 | I | 3.60 | 865 | 3.89 | 2245 | 14754 | 3.22 | 1.46 | 157 | 538 | 0.004 | 3.02 | 0.12 | 2.17 | 6.67 | 0.29 | 41.0 | 17.1 | 211 | 73.4 | 350 | 66.8 | 473 | 88.0 | 664 |
| 13 | III | bdl | 39.5 | 0.67 | 415 | 16844 | 2.98 | 1.27 | 1.62 | 385 | bdl | 0.13 | bdl | bdl | 0.11 | 0.039 | 1.11 | 1.01 | 21.6 | 10.9 | 58.6 | 11.9 | 85.5 | 16.5 | 543 |
| 14 | III | 5.33 | 22.9 | 0.35 | 99.9 | 17786 | 1.48 | 1.05 | 0.72 | 109 | bdl | 0.041 | 0.004 | bdl | 0.12 | 0.006 | 0.55 | 0.23 | 4.77 | 2.34 | 14.1 | 3.12 | 24.5 | 4.98 | 505 |
| 15 | III | 1.02 | 19.6 | 0.35 | 195 | 17575 | 2.34 | 0.88 | 0.61 | 128 | bdl | 0.048 | bdl | bdl | bdl | 0.014 | 0.51 | 0.28 | 8.75 | 4.73 | 31.7 | 7.96 | 73.9 | 17.6 | 505 |
| 16 | I | 4.44 | 1342 | 7.01 | 3481 | 11850 | 5.06 | 1.39 | 263 | 569 | bdl | 5.88 | 0.15 | 3.05 | 10.5 | 0.52 | 62.4 | 24.0 | 315 | 118 | 605 | 119 | 878 | 171 | 714 |
| 17 | I | 2.84 | 1358 | 2.50 | 2873 | 13927 | 5.58 | 2.22 | 169 | 708 | 0.005 | 2.82 | 0.064 | 0.96 | 4.78 | 0.20 | 37.8 | 18.2 | 248 | 92.8 | 478 | 96.0 | 698 | 136 | 630 |
| 18 | II | 2.20 | 37.7 | 0.20 | 273 | 13981 | 2.42 | 1.12 | 1.26 | 223 | bdl | 0.067 | bdl | bdl | bdl | 0.005 | 0.20 | 0.24 | 8.51 | 6.19 | 66.8 | 23.6 | 253 | 64.0 | 476 |
| 19 | III | 1.73 | 33.5 | 0.50 | 304 | 18742 | 1.57 | 0.56 | 0.72 | 130 | bdl | 0.068 | bdl | 0.034 | bdl | 0.007 | 0.60 | 0.71 | 15.4 | 7.67 | 42.3 | 7.87 | 56.8 | 10.1 | 525 |
| 20 | I | 4.89 | 790 | 4.50 | 1985 | 12358 | 4.53 | 1.47 | 178 | 392 | 0.009 | 4.18 | 0.11 | 2.46 | 5.84 | 0.28 | 36.1 | 14.4 | 178 | 67.1 | 346 | 69.4 | 515 | 104 | 676 |
| 21 | I | 1.31 | 1220 | 2.60 | 2609 | 14463 | 4.46 | 1.79 | 104 | 507 | bdl | 2.21 | 0.040 | 1.33 | 4.52 | 0.24 | 34.7 | 15.1 | 215 | 83.5 | 437 | 89.4 | 667 | 135 | 633 |
| 22 | I | bdl | 749 | 13.2 | 2020 | 10382 | 2.95 | 1.04 | 115 | 283 | bdl | 3.03 | 0.13 | 1.98 | 6.39 | 0.44 | 33.0 | 13.0 | 174 | 66.0 | 341 | 69.7 | 522 | 109 | 772 |
| 23 | I | 25.8 | 1455 | 6.80 | 3422 | 12611 | 6.67 | 1.80 | 231 | 586 | 0.013 | 4.91 | 0.13 | 2.85 | 8.84 | 0.38 | 57.9 | 23.1 | 303 | 113 | 586 | 117 | 865 | 173 | 711 |
| 24 | III | 1.22 | 42.4 | 0.29 | 160 | 17905 | 1.19 | 0.52 | 0.75 | 94.9 | bdl | 0.017 | bdl | bdl | 0.10 | 0.006 | 0.41 | 0.34 | 6.60 | 3.84 | 26.4 | 6.17 | 47.7 | 10.8 | 495 |
| 25 | I | 5.30 | 644 | 5.85 | 1145 | 13427 | 1.85 | 0.87 | 71.0 | 222 | 0.013 | 1.99 | 0.032 | 1.19 | 3.18 | 0.20 | 21.0 | 7.59 | 101 | 37.2 | 196 | 40.5 | 304 | 63.6 | 698 |
| 26 | III | 2.13 | 23.6 | 0.19 | 195 | 16470 | 1.52 | 0.88 | 0.74 | 152 | bdl | 0.08 | bdl | bdl | bdl | bdl | 0.40 | 0.33 | 8.71 | 4.64 | 32.2 | 8.34 | 71.7 | 17.2 | 473 |
| 27 | I | 14.2 | 2685 | 2.71 | 4870 | 20103 | 8.62 | 5.05 | 169 | 2677 | bdl | 1.27 | 0.022 | 0.74 | 3.98 | 0.086 | 43.1 | 25.2 | 406 | 160 | 917 | 214 | 1755 | 365 | 636 |
| 28 | I | 0.94 | 392 | 6.24 | 738 | 10155 | 1.15 | 0.41 | 32.0 | 91.6 | 0.009 | 1.21 | 0.043 | 0.97 | 2.89 | 0.26 | 14.1 | 5.13 | 64.7 | 24.5 | 126 | 25.9 | 200 | 41.8 | 703 |

Table S5 (continued)

| No. | Group | Al | P | Ti | Y | Hf | Nb | Ta | Th | U | La | Ce | Pr | Nd | Sm | Eu | Gd | Tb | Dy | Ho | Er | Tm | Yb | Lu | T/^o^C |
| --- | --- | --- | --- | --- | --- | --- | --- | --- | --- | --- | --- | --- | --- | --- | --- | --- | --- | --- | --- | --- | --- | --- | --- | --- | --- |
| 29 | I | 5.13 | 1164 | 3.38 | 2180 | 14231 | 6.60 | 2.14 | 145 | 774 | bdl | 8.20 | 0.049 | 1.17 | 3.64 | 0.34 | 29.9 | 12.9 | 181 | 68.3 | 350 | 69.6 | 512 | 103 | 653 |
| 30 | I | 21.0 | 931 | 6.08 | 2013 | 12563 | 2.10 | 0.76 | 116 | 306 | 0.018 | 4.11 | 0.14 | 1.84 | 6.53 | 0.27 | 35.4 | 13.4 | 174 | 67.7 | 360 | 73.1 | 550 | 114 | 701 |

Note: All the trace elements are in unit of µg/g. Group I, relict magmatic domains; Group II, metamorphic domains with steep HREE patterns (Rim-I); Group III, metamorphic domains with flatterned HREE patterns (Rim-II). The Ti-in-zircon temperature was calculated following the calibration of Ferry and Watson (2007) assuming the saturation of quartz and rutile. “bdl”, below the detection limit.

Table S6. Major elements in garnet, jadeite, muscovite, albite and epidote from orthogneiss 13AP41

| No. | G1-1 | G1-2 | G1-3 | G1-4 | G1-5 | G1-6 | G1-7 | G1-8 | G1-9 | G1-10 | G1-11 | G1-12 | G1-13 | G1-14 | G1-15 | G1-16 |
| --- | --- | --- | --- | --- | --- | --- | --- | --- | --- | --- | --- | --- | --- | --- | --- | --- |
| SiO_2_ | 37.63 | 37.66 | 37.96 | 37.79 | 38.26 | 38.09 | 38.08 | 38.28 | 38.29 | 38.18 | 38.04 | 37.83 | 38.06 | 38.23 | 38.19 | 38.12 |
| TiO_2_ | 0.03 | 0.03 | 0.04 | 0.04 | 0.06 | 0.06 | 0.05 | 0.08 | 0.07 | 0.07 | 0.03 | 0.02 | 0.06 | 0.04 | 0.06 | 0.08 |
| Al_2_O_3_ | 21.21 | 21.21 | 21.37 | 21.39 | 21.29 | 21.30 | 21.17 | 21.14 | 21.29 | 21.33 | 21.36 | 21.58 | 21.42 | 21.36 | 21.33 | 21.35 |
| FeO | 20.84 | 21.99 | 22.30 | 22.43 | 17.71 | 17.91 | 17.41 | 18.00 | 17.54 | 17.57 | 22.16 | 23.04 | 18.43 | 17.98 | 17.98 | 18.61 |
| MnO | 3.94 | 2.45 | 2.37 | 2.43 | 3.35 | 3.22 | 3.43 | 3.43 | 3.36 | 3.16 | 2.41 | 2.32 | 3.21 | 3.28 | 3.32 | 3.11 |
| MgO | 0.21 | 0.28 | 0.30 | 0.29 | 0.23 | 0.22 | 0.22 | 0.25 | 0.25 | 0.23 | 0.27 | 0.31 | 0.23 | 0.22 | 0.23 | 0.24 |
| CaO | 16.01 | 16.25 | 16.03 | 15.89 | 19.12 | 19.08 | 19.21 | 19.18 | 19.22 | 19.39 | 16.02 | 15.72 | 18.96 | 19.14 | 18.95 | 18.68 |
| Na_2_O | 0.03 | 0.04 | 0.05 | 0.06 | 0.05 | 0.07 | 0.08 | 0.07 | 0.06 | 0.05 | 0.04 | 0.04 | 0.05 | 0.06 | 0.05 | 0.06 |
| Cr_2_O_3_ | 0.02 | bdl | bdl | bdl | 0.01 | bdl | 0.02 | 0.01 | bdl | 0.03 | bdl | bdl | bdl | bdl | bdl | bdl |
| NiO | bdl | bdl | bdl | bdl | 0.01 | bdl | bdl | 0.01 | 0.01 | bdl | 0.03 | bdl | bdl | bdl | bdl | bdl |
| Total | 99.92 | 99.91 | 100.42 | 100.32 | 100.09 | 99.96 | 99.67 | 100.45 | 100.09 | 100.00 | 100.36 | 100.87 | 100.42 | 100.31 | 100.12 | 100.24 |
| O = 12 |  |  |  |  |  |  |  |  |  |  |  |  |  |  |  |  |
| Si | 2.986 | 2.986 | 2.993 | 2.985 | 3.002 | 2.995 | 3.000 | 2.997 | 3.003 | 2.997 | 3.000 | 2.976 | 2.983 | 2.995 | 2.998 | 2.992 |
| Al^iv^ | 0.014 | 0.014 | 0.007 | 0.015 | - | 0.005 | - | 0.003 | - | 0.003 | - | 0.024 | 0.017 | 0.005 | 0.002 | 0.008 |
| Al^vi^ | 1.971 | 1.970 | 1.981 | 1.979 | 1.971 | 1.971 | 1.968 | 1.950 | 1.970 | 1.972 | 1.986 | 1.978 | 1.964 | 1.970 | 1.973 | 1.969 |
| Ti | 0.002 | 0.002 | 0.002 | 0.002 | 0.004 | 0.004 | 0.003 | 0.005 | 0.004 | 0.004 | 0.002 | 0.001 | 0.003 | 0.002 | 0.004 | 0.005 |
| Cr | 0.002 | - | - | - | 0.001 | - | 0.001 | 0.001 | - | 0.002 | - | - | - | - | - | - |
| Fe^3+^ | 0.023 | 0.025 | 0.015 | 0.016 | 0.020 | 0.023 | 0.025 | 0.039 | 0.021 | 0.019 | 0.011 | 0.018 | 0.029 | 0.024 | 0.020 | 0.023 |
| Fe^2+^ | 1.360 | 1.433 | 1.455 | 1.466 | 1.143 | 1.155 | 1.123 | 1.139 | 1.130 | 1.134 | 1.451 | 1.497 | 1.179 | 1.154 | 1.160 | 1.198 |
| Mn | 0.265 | 0.164 | 0.158 | 0.162 | 0.222 | 0.215 | 0.229 | 0.228 | 0.223 | 0.210 | 0.161 | 0.155 | 0.213 | 0.218 | 0.221 | 0.207 |
| Mg | 0.025 | 0.033 | 0.036 | 0.034 | 0.027 | 0.026 | 0.026 | 0.029 | 0.029 | 0.027 | 0.031 | 0.036 | 0.027 | 0.026 | 0.027 | 0.028 |
| Ni | - | - | - | - | - | - | - | 0.001 | 0.001 | - | 0.002 | - | - | - | - | - |
| Ca | 1.361 | 1.381 | 1.354 | 1.345 | 1.607 | 1.607 | 1.622 | 1.609 | 1.615 | 1.631 | 1.354 | 1.325 | 1.592 | 1.607 | 1.594 | 1.571 |
| Na | 0.002 | 0.003 | 0.004 | 0.005 | 0.004 | 0.006 | 0.006 | 0.006 | 0.005 | 0.004 | 0.004 | 0.004 | 0.004 | 0.005 | 0.004 | 0.005 |
| Total | 8.010 | 8.011 | 8.005 | 8.009 | 8.001 | 8.005 | 8.003 | 8.007 | 8.000 | 8.003 | 8.001 | 8.015 | 8.012 | 8.006 | 8.003 | 8.006 |
| Almandine | 44.7 | 47.1 | 48.3 | 48.4 | 37.8 | 38.3 | 37.2 | 37.5 | 37.4 | 37.6 | 48.3 | 49.0 | 38.6 | 38.2 | 38.4 | 39.7 |
| Andradite | 1.1 | 1.3 | 0.8 | 0.8 | 1.0 | 1.1 | 1.2 | 2.0 | 1.0 | 1.0 | 0.5 | 0.9 | 1.5 | 1.2 | 1.0 | 1.2 |
| Grossular | 44.4 | 45.0 | 44.5 | 44.2 | 52.8 | 52.5 | 53.0 | 51.9 | 53.1 | 53.4 | 44.7 | 43.6 | 51.9 | 52.4 | 52.2 | 51.3 |
| Pyrope | 0.8 | 1.1 | 1.2 | 1.1 | 0.9 | 0.9 | 0.9 | 1.0 | 1.0 | 0.9 | 1.1 | 1.2 | 0.9 | 0.9 | 0.9 | 0.9 |
| Spessartine | 8.9 | 5.5 | 5.3 | 5.4 | 7.5 | 7.2 | 7.7 | 7.6 | 7.5 | 7.0 | 5.4 | 5.2 | 7.1 | 7.3 | 7.4 | 6.9 |
| Uvarovite | 0.1 | 0.0 | 0.0 | 0.0 | 0.0 | 0.0 | 0.1 | 0.0 | 0.0 | 0.1 | 0.0 | 0.0 | 0.0 | 0.0 | 0.0 | 0.0 |

Table S6 (continued)

| No. | G2-1 | G2-2 | G2-3 | G2-4 | G2-5 | G2-6 | G2-7 | G2-8 | G2-9 | G2-10 | G2-11 | G2-12 | G2-13 | G2-14 | G2-15 | G2-16 |
| --- | --- | --- | --- | --- | --- | --- | --- | --- | --- | --- | --- | --- | --- | --- | --- | --- |
| SiO_2_ | 38.14 | 37.90 | 37.79 | 37.89 | 38.07 | 37.97 | 37.77 | 37.79 | 38.15 | 38.15 | 38.09 | 38.01 | 38.17 | 37.93 | 37.70 | 38.03 |
| TiO_2_ | 0.04 | 0.03 | 0.04 | 0.04 | 0.06 | 0.06 | 0.03 | 0.05 | 0.04 | 0.07 | 0.06 | 0.05 | 0.06 | 0.03 | 0.02 | 0.07 |
| Al_2_O_3_ | 21.27 | 21.31 | 21.52 | 21.35 | 21.37 | 21.49 | 21.58 | 21.32 | 21.35 | 21.41 | 21.36 | 21.30 | 21.29 | 21.37 | 21.39 | 21.26 |
| FeO | 21.96 | 22.45 | 22.44 | 21.71 | 19.58 | 19.87 | 22.37 | 22.54 | 20.09 | 16.89 | 17.88 | 17.75 | 18.48 | 20.99 | 22.66 | 17.07 |
| MnO | 2.49 | 2.30 | 2.32 | 2.47 | 2.76 | 2.90 | 2.28 | 2.36 | 2.76 | 3.16 | 3.32 | 3.32 | 3.09 | 3.46 | 2.33 | 3.86 |
| MgO | 0.28 | 0.29 | 0.29 | 0.27 | 0.25 | 0.28 | 0.29 | 0.32 | 0.26 | 0.23 | 0.22 | 0.22 | 0.23 | 0.21 | 0.31 | 0.23 |
| CaO | 16.06 | 16.20 | 16.11 | 16.79 | 18.20 | 17.82 | 16.19 | 15.87 | 17.67 | 20.15 | 19.12 | 19.27 | 18.81 | 16.46 | 16.06 | 19.41 |
| Na_2_O | 0.03 | 0.04 | 0.04 | 0.07 | 0.06 | 0.07 | 0.04 | 0.05 | 0.08 | 0.08 | 0.07 | 0.04 | 0.06 | 0.03 | 0.02 | 0.09 |
| Cr_2_O_3_ | 0.02 | 0.01 | bdl | 0.02 | 0.02 | 0.02 | 0.01 | 0.01 | 0.03 | 0.01 | 0.01 | 0.01 | bdl | 0.01 | 0.01 | bdl |
| NiO | bdl | 0.01 | 0.01 | bdl | 0.02 | 0.01 | bdl | 0.02 | bdl | bdl | bdl | bdl | bdl | bdl | bdl | 0.01 |
| Total | 100.30 | 100.54 | 100.56 | 100.61 | 100.40 | 100.49 | 100.56 | 100.33 | 100.43 | 100.15 | 100.13 | 99.98 | 100.20 | 100.50 | 100.50 | 100.02 |
| O = 12 |  |  |  |  |  |  |  |  |  |  |  |  |  |  |  |  |
| Si | 3.008 | 2.987 | 2.978 | 2.981 | 2.988 | 2.981 | 2.976 | 2.985 | 2.996 | 2.987 | 2.990 | 2.988 | 2.996 | 2.989 | 2.975 | 2.988 |
| Al^iv^ | - | 0.013 | 0.022 | 0.019 | 0.012 | 0.019 | 0.024 | 0.015 | 0.004 | 0.013 | 0.010 | 0.012 | 0.004 | 0.011 | 0.025 | 0.012 |
| Al^vi^ | 1.978 | 1.969 | 1.979 | 1.964 | 1.968 | 1.972 | 1.981 | 1.973 | 1.974 | 1.965 | 1.968 | 1.964 | 1.968 | 1.975 | 1.968 | 1.959 |
| Ti | 0.002 | 0.002 | 0.002 | 0.002 | 0.004 | 0.004 | 0.002 | 0.003 | 0.003 | 0.004 | 0.004 | 0.003 | 0.003 | 0.002 | 0.001 | 0.004 |
| Cr | 0.001 | 0.001 | - | 0.001 | 0.001 | 0.001 | - | 0.001 | 0.002 | 0.001 | 0.001 | - | - | 0.001 | 0.001 | - |
| Fe^3+^ | 0.010 | 0.025 | 0.017 | 0.029 | 0.024 | 0.021 | 0.015 | 0.021 | 0.020 | 0.026 | 0.024 | 0.028 | 0.025 | 0.020 | 0.027 | 0.033 |
| Fe^2+^ | 1.439 | 1.454 | 1.462 | 1.400 | 1.261 | 1.284 | 1.459 | 1.468 | 1.300 | 1.080 | 1.150 | 1.139 | 1.188 | 1.363 | 1.468 | 1.089 |
| Mn | 0.167 | 0.153 | 0.155 | 0.165 | 0.184 | 0.193 | 0.152 | 0.158 | 0.184 | 0.210 | 0.221 | 0.221 | 0.206 | 0.231 | 0.156 | 0.257 |
| Mg | 0.033 | 0.034 | 0.035 | 0.031 | 0.029 | 0.033 | 0.034 | 0.038 | 0.030 | 0.026 | 0.026 | 0.026 | 0.027 | 0.025 | 0.036 | 0.026 |
| Ni | - | - | 0.001 | - | 0.001 | 0.001 | - | 0.001 | - | - | - | - | - | - | - | - |
| Ca | 1.357 | 1.368 | 1.361 | 1.416 | 1.531 | 1.499 | 1.367 | 1.344 | 1.487 | 1.691 | 1.608 | 1.624 | 1.582 | 1.389 | 1.358 | 1.634 |
| Na | 0.003 | 0.003 | 0.003 | 0.006 | 0.005 | 0.006 | 0.004 | 0.005 | 0.006 | 0.006 | 0.006 | 0.004 | 0.005 | 0.003 | 0.002 | 0.008 |
| Total | 7.997 | 8.011 | 8.013 | 8.014 | 8.009 | 8.012 | 8.014 | 8.010 | 8.005 | 8.010 | 8.008 | 8.010 | 8.005 | 8.009 | 8.017 | 8.011 |
| Almandine | 47.8 | 47.9 | 47.9 | 45.9 | 41.6 | 42.2 | 47.8 | 48.4 | 43.2 | 35.5 | 38.0 | 37.4 | 39.3 | 44.9 | 47.9 | 35.8 |
| Andradite | 0.5 | 1.3 | 0.9 | 1.4 | 1.2 | 1.0 | 0.7 | 1.1 | 1.0 | 1.3 | 1.2 | 1.4 | 1.3 | 1.0 | 1.4 | 1.6 |
| Grossular | 45.0 | 44.5 | 44.8 | 46.0 | 50.0 | 49.2 | 45.2 | 43.9 | 48.6 | 55.2 | 52.5 | 52.9 | 51.6 | 45.4 | 44.2 | 53.1 |
| Pyrope | 1.1 | 1.2 | 1.2 | 1.0 | 1.0 | 1.1 | 1.1 | 1.3 | 1.0 | 0.9 | 0.9 | 0.9 | 0.9 | 0.8 | 1.2 | 0.9 |
| Spessartine | 5.6 | 5.1 | 5.2 | 5.5 | 6.2 | 6.5 | 5.1 | 5.3 | 6.1 | 7.0 | 7.4 | 7.4 | 6.9 | 7.7 | 5.2 | 8.6 |
| Uvarovite | 0.1 | 0.0 | 0.0 | 0.0 | 0.1 | 0.0 | 0.0 | 0.0 | 0.1 | 0.0 | 0.0 | 0.0 | 0.0 | 0.0 | 0.0 | 0.0 |

Table S6 (continued)

| No. | Jd1-1 | Jd1-2 | Jd2-1 | Jd2-2 | Jd2-3 | Jd3-1 | Jd3-2 |
| --- | --- | --- | --- | --- | --- | --- | --- |
| SiO_2_ | 58.73 | 58.87 | 59.07 | 58.50 | 58.26 | 59.08 | 59.04 |
| TiO_2_ | 0.01 | 0.01 | 0.02 | 0.02 | 0.02 | 0.01 | 0.02 |
| Al_2_O_3_ | 22.50 | 22.48 | 22.30 | 21.94 | 21.82 | 22.09 | 22.15 |
| FeO | 3.34 | 3.54 | 3.30 | 3.21 | 3.49 | 3.82 | 3.51 |
| MnO | 0.02 | 0.03 | 0.00 | 0.02 | 0.05 | 0.03 | 0.05 |
| MgO | 0.46 | 0.38 | 0.36 | 0.60 | 0.54 | 0.39 | 0.54 |
| CaO | 1.81 | 1.22 | 1.19 | 2.21 | 1.94 | 1.13 | 1.91 |
| Na_2_O | 13.60 | 13.81 | 13.88 | 13.36 | 13.50 | 13.80 | 13.26 |
| Cr_2_O_3_ | bdl | 0.04 | 0.01 | bdl | bdl | 0.01 | 0.01 |
| NiO | bdl | bdl | bdl | 0.01 | bdl | bdl | 0.01 |
| Total | 100.46 | 100.38 | 100.13 | 99.87 | 99.62 | 100.35 | 100.50 |
| O = 6; Cation = 4 | | | | | | | |
| Si | 2.007 | 2.012 | 2.022 | 2.013 | 2.009 | 2.022 | 2.023 |
| Ti | - | - | - | 0.001 | - | - | - |
| Al | 0.906 | 0.905 | 0.900 | 0.890 | 0.887 | 0.891 | 0.895 |
| Cr | - | 0.001 | - | - | - | - | - |
| Fe | 0.095 | 0.101 | 0.094 | 0.092 | 0.101 | 0.109 | 0.101 |
| Mn | 0.001 | 0.001 | - | - | 0.002 | 0.001 | 0.001 |
| Mg | 0.023 | 0.019 | 0.019 | 0.031 | 0.028 | 0.020 | 0.028 |
| Ca | 0.066 | 0.045 | 0.044 | 0.082 | 0.072 | 0.041 | 0.070 |
| Na | 0.901 | 0.915 | 0.921 | 0.891 | 0.903 | 0.916 | 0.881 |
| Jadeite | 93.2 | 95.3 | 95.5 | 91.6 | 92.7 | 95.7 | 92.6 |
| Diopside | 6.8 | 4.7 | 4.5 | 8.4 | 7.3 | 4.3 | 7.4 |

Table S6 (continued)

| No. | Ms1-1 | Ms1-2 | Ms2-1 | Ms2-2 | Ms3-1 | Ms3-2 | Ms4-1 | Ms5-1 | Ms5-2 | Ms6-1 | Ms7-1 | Ms7-2 |
| --- | --- | --- | --- | --- | --- | --- | --- | --- | --- | --- | --- | --- |
| SiO_2_ | 49.04 | 49.00 | 49.08 | 48.69 | 49.23 | 49.01 | 48.43 | 48.66 | 48.80 | 48.63 | 47.91 | 48.91 |
| TiO_2_ | 0.39 | 0.39 | 0.15 | 0.42 | 0.42 | 0.37 | 0.46 | 0.38 | 0.47 | 0.30 | 0.46 | 0.35 |
| Al_2_O_3_ | 30.47 | 30.44 | 29.09 | 31.36 | 31.15 | 30.46 | 31.15 | 31.57 | 31.13 | 31.52 | 31.01 | 29.77 |
| FeO | 4.13 | 4.38 | 5.42 | 3.97 | 3.95 | 4.18 | 3.89 | 3.94 | 4.19 | 3.64 | 3.74 | 4.39 |
| MnO | 0.04 | bdl | 0.02 | 0.02 | 0.05 | 0.03 | 0.05 | 0.04 | 0.05 | 0.05 | 0.01 | 0.01 |
| MgO | 1.37 | 1.27 | 1.46 | 1.26 | 1.35 | 1.34 | 1.28 | 1.28 | 1.17 | 1.25 | 1.28 | 1.38 |
| CaO | 0.01 | bdl | 0.01 | 0.01 | bdl | 0.01 | 0.01 | 0.03 | 0.01 | bdl | 0.06 | 0.03 |
| Na_2_O | 0.32 | 0.20 | 0.31 | 0.44 | 0.47 | 0.26 | 0.39 | 0.46 | 0.27 | 0.48 | 0.44 | 0.22 |
| K_2_O | 10.58 | 10.72 | 10.80 | 10.58 | 10.66 | 10.90 | 10.49 | 10.54 | 10.88 | 10.66 | 10.43 | 10.60 |
| Cr_2_O_3_ | 0.01 | 0.01 | 0.02 | bdl | 0.01 | bdl | bdl | bdl | bdl | bdl | 0.04 | 0.06 |
| NiO | bdl | 0.02 | bdl | 0.01 | 0.01 | bdl | 0.01 | bdl | 0.01 | 0.01 | 0.01 | bdl |
| Total | 96.37 | 96.43 | 96.34 | 96.76 | 97.29 | 96.57 | 96.15 | 96.91 | 96.98 | 96.54 | 95.39 | 95.73 |
| O = 11 | | | | | | | | | | | | |
| Si | 3.266 | 3.266 | 3.297 | 3.229 | 3.247 | 3.264 | 3.230 | 3.221 | 3.237 | 3.229 | 3.222 | 3.285 |
| Al^iv^ | 0.734 | 0.734 | 0.703 | 0.771 | 0.753 | 0.736 | 0.770 | 0.779 | 0.763 | 0.771 | 0.778 | 0.715 |
| Al^vi^ | 1.659 | 1.658 | 1.601 | 1.680 | 1.668 | 1.655 | 1.679 | 1.685 | 1.670 | 1.696 | 1.680 | 1.641 |
| Ti | 0.020 | 0.019 | 0.007 | 0.021 | 0.021 | 0.019 | 0.023 | 0.019 | 0.023 | 0.015 | 0.023 | 0.018 |
| Cr | 0.001 | 0.001 | 0.001 | - | - | - | - | - | - | - | 0.002 | 0.003 |
| Fe | 0.230 | 0.244 | 0.304 | 0.220 | 0.218 | 0.233 | 0.217 | 0.218 | 0.233 | 0.202 | 0.210 | 0.246 |
| Mn | 0.002 | - | 0.001 | 0.001 | 0.003 | 0.002 | 0.003 | 0.002 | 0.003 | 0.003 | 0.001 | 0.001 |
| Mg | 0.136 | 0.127 | 0.146 | 0.125 | 0.133 | 0.133 | 0.128 | 0.127 | 0.116 | 0.123 | 0.128 | 0.139 |
| Ca | 0.001 | - | 0.001 | 0.001 | - | - | 0.001 | 0.002 | 0.001 | - | 0.004 | 0.002 |
| Na | 0.041 | 0.026 | 0.040 | 0.056 | 0.060 | 0.034 | 0.050 | 0.059 | 0.035 | 0.061 | 0.058 | 0.029 |
| K | 0.899 | 0.911 | 0.925 | 0.895 | 0.897 | 0.926 | 0.892 | 0.890 | 0.921 | 0.903 | 0.895 | 0.908 |
| Mg/Fe+Mg | 0.37 | 0.34 | 0.32 | 0.36 | 0.38 | 0.36 | 0.37 | 0.37 | 0.33 | 0.38 | 0.38 | 0.36 |

Table S6 (continued)

| No. | Ab-1 | Ab-2 | Ab-3 | Ab-4 |
| --- | --- | --- | --- | --- |
| SiO_2_ | 68.55 | 69.36 | 68.83 | 69.17 |
| TiO_2_ | bdl | bdl | 0.01 | 0.02 |
| Al_2_O_3_ | 19.18 | 19.36 | 18.92 | 19.22 |
| FeO | 0.36 | 0.33 | 0.34 | 0.28 |
| MnO | 0.03 | bdl | 0.08 | 0.03 |
| MgO | bdl | bdl | 0.01 | bdl |
| CaO | 0.09 | 0.06 | 0.11 | 0.15 |
| Na_2_O | 11.79 | 11.72 | 11.68 | 11.69 |
| K_2_O | 0.05 | 0.03 | 0.02 | 0.03 |
| Total | 100.04 | 100.86 | 99.99 | 100.59 |
| Cations = 5 | | | | |
| Si | 2.994 | 3.009 | 3.011 | 3.008 |
| Ti | - | - | - | 0.001 |
| Al | 0.987 | 0.990 | 0.976 | 0.985 |
| Fe | 0.013 | 0.012 | 0.012 | 0.010 |
| Mn | 0.001 | - | 0.003 | 0.001 |
| Ca | 0.004 | 0.003 | 0.005 | 0.007 |
| Na | 0.998 | 0.986 | 0.991 | 0.986 |
| K | 0.003 | 0.001 | 0.001 | 0.002 |
| An | 0.42 | 0.291 | 0.51 | 0.69 |
| Ab | 99.30 | 99.569 | 99.40 | 99.14 |
| Or | 0.28 | 0.140 | 0.09 | 0.17 |

Table S6 (continued)

| No. | Ep-1 | Ep-2 | Ep-3 | Ep-4 | Ep-5 | Ep-6 | Ep-7 | Ep-8 |
| --- | --- | --- | --- | --- | --- | --- | --- | --- |
| SiO_2_ | 38.19 | 38.82 | 38.73 | 38.69 | 38.00 | 38.81 | 37.90 | 38.90 |
| TiO_2_ | 0.09 | 0.10 | 0.11 | 0.14 | 0.13 | 0.06 | 0.11 | 0.13 |
| Al_2_O_3_ | 28.10 | 28.41 | 28.30 | 28.14 | 23.77 | 28.22 | 27.68 | 26.42 |
| FeO | 5.55 | 6.06 | 5.88 | 6.29 | 11.55 | 6.61 | 6.20 | 8.50 |
| MnO | 0.10 | 0.06 | 0.07 | 0.05 | 0.07 | 0.11 | 0.08 | 0.05 |
| MgO | 0.01 | 0.00 | 0.05 | 0.01 | 0.02 | 0.00 | 0.00 | 0.01 |
| CaO | 22.19 | 23.69 | 22.52 | 23.75 | 23.61 | 23.46 | 22.81 | 23.66 |
| Na_2_O | 0.08 | 0.01 | 0.03 | 0.01 | 0.00 | 0.02 | 0.00 | 0.04 |
| K_2_O | 0.03 | 0.00 | 0.00 | 0.00 | 0.00 | 0.02 | 0.00 | 0.00 |
| Total | 94.35 | 97.15 | 95.70 | 97.07 | 97.15 | 97.31 | 94.78 | 97.70 |
| O=12.5 | | | | | | | | |
| Si | 3.038 | 3.013 | 3.039 | 3.010 | 3.012 | 3.011 | 3.014 | 3.025 |
| Ti | 0.006 | 0.006 | 0.007 | 0.008 | 0.008 | 0.003 | 0.006 | 0.007 |
| Al | 2.635 | 2.600 | 2.618 | 2.581 | 2.222 | 2.581 | 2.595 | 2.422 |
| Fe^3+^ | 0.366 | 0.389 | 0.382 | 0.397 | 0.694 | 0.425 | 0.408 | 0.541 |
| Fe^2+^ | 0.004 | 0.004 | 0.004 | 0.012 | 0.071 | 0.004 | 0.004 | 0.012 |
| Mn | 0.007 | 0.004 | 0.005 | 0.003 | 0.004 | 0.007 | 0.005 | 0.003 |
| Mg | 0.001 | 0.000 | 0.005 | 0.001 | 0.002 | 0.000 | 0.000 | 0.001 |
| Ca | 1.892 | 1.970 | 1.893 | 1.980 | 2.006 | 1.950 | 1.943 | 1.972 |
| Na | 0.012 | 0.002 | 0.004 | 0.001 | 0.000 | 0.003 | 0.000 | 0.006 |
| K | 0.003 | 0.000 | 0.000 | 0.000 | 0.000 | 0.002 | 0.000 | 0.000 |
| Sum | 7.963 | 7.988 | 7.957 | 7.993 | 8.023 | 7.986 | 7.978 | 7.989 |

Note: The major oxides in the minerals are in unit of wt.%. G1 and G2 are garnet grains Grt-1 and Grt-2, respectively. bdl, below the detection limit. The Fe^3+^ in garnet was calculated assuming full site occupancy. Mineral abbreviations: Jd, jadeite; Ms, muscovite; Ab, albite; Ep, epidote.

**3. Supplemental Figures**


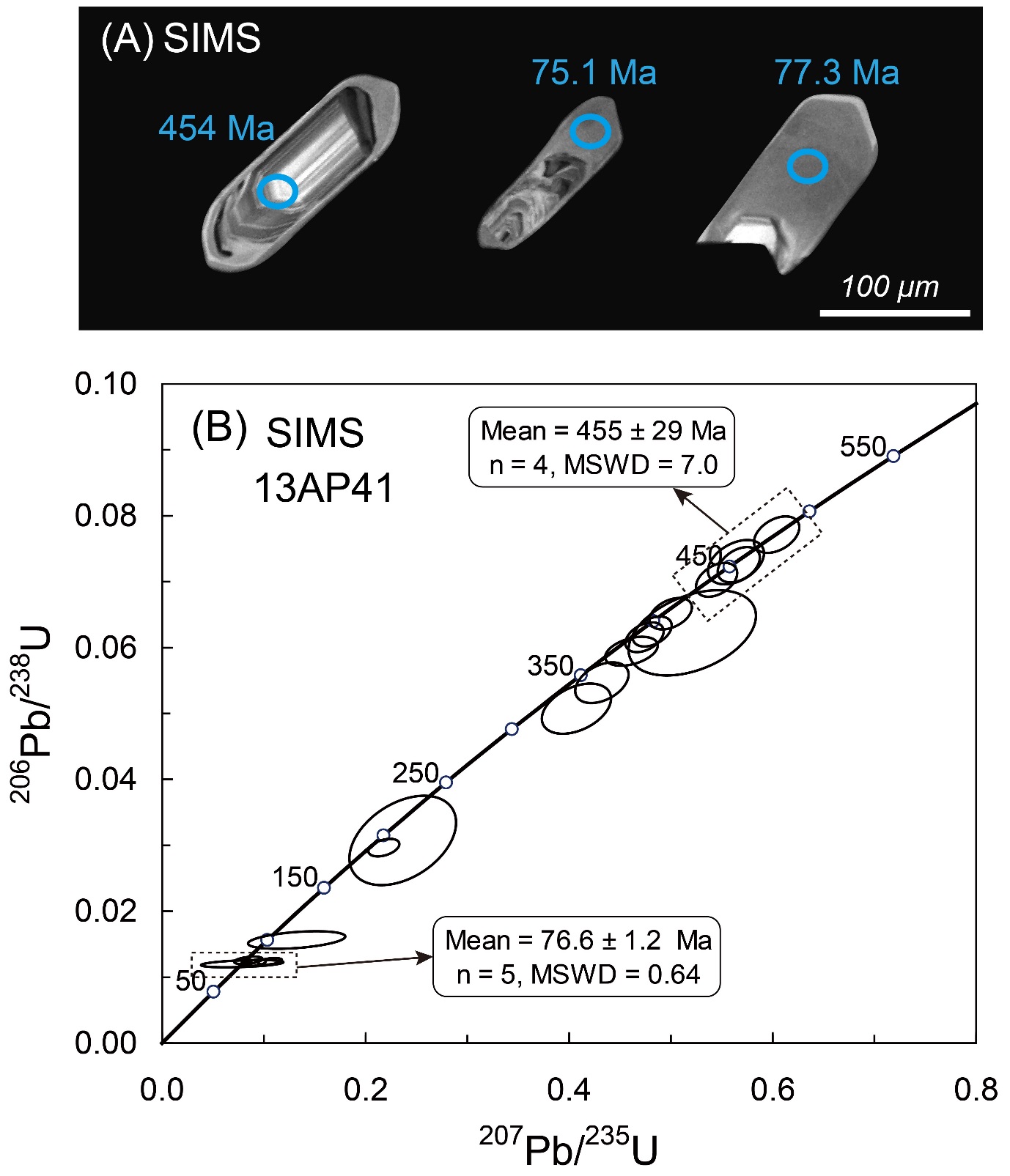


Figure S1. Zircon CL images (a) and U-Pb concordia diagram (b) of the orthogneiss 13AP41. The ^206^Pb/^238^U age in panel (a) are from SIMS analysis.


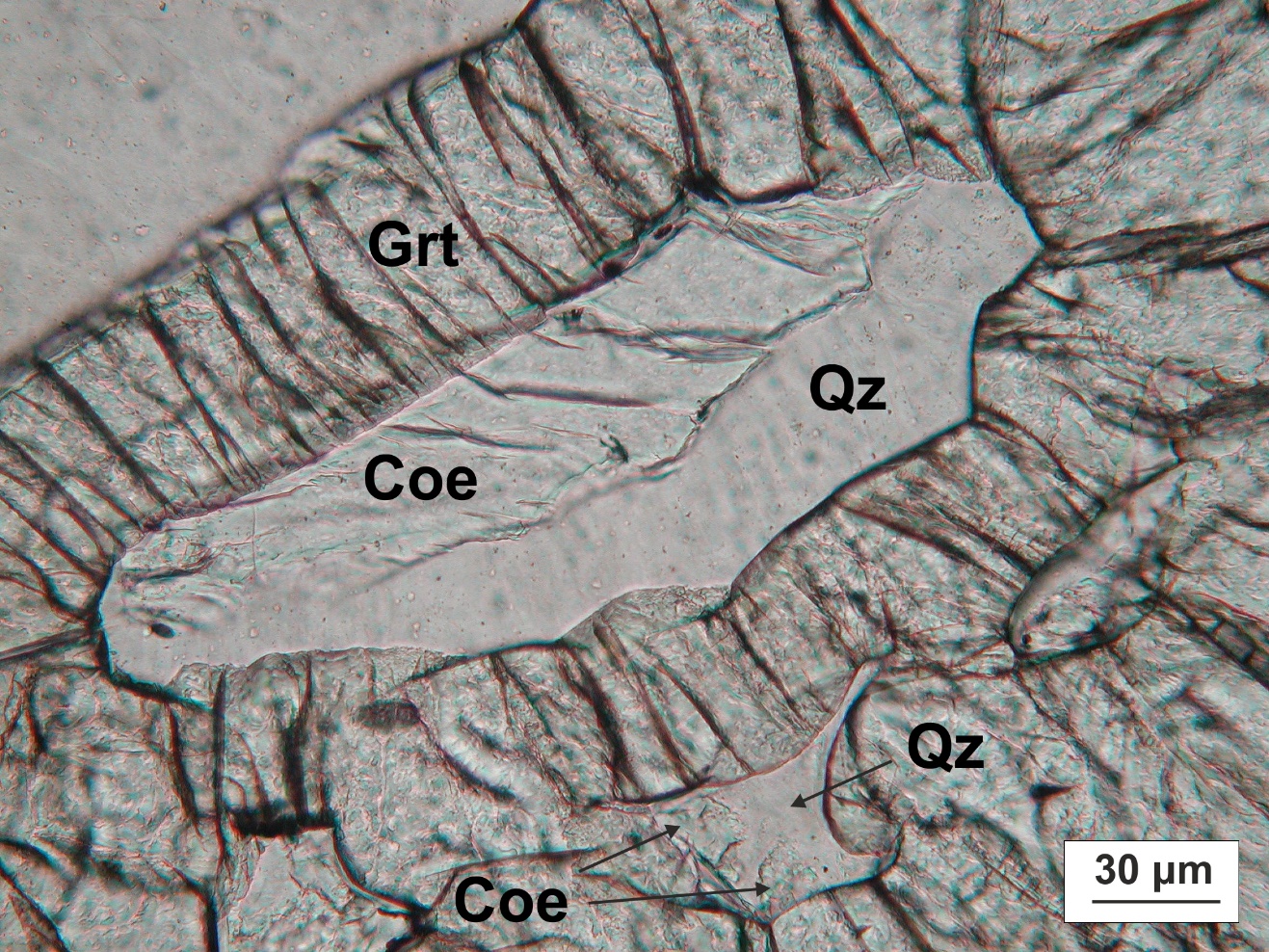


Figure S2. Coesite and coexisting quartz inclusions in the pyrope garnet of whiteschist from the Dora-Maira Massif. Note that the coesite relics occurred at the edge of quartz grains. Grt, garnet; Coe, Coesite; Qz, quartz. The photo is taken under plane-polarized light.

**References in the Supplemental Materials**

Chen, Y. X., Lu, W. N., He, Y. S., Schertl, H. P., Zheng, Y. F., Xiong, J. W., and Zhou, K., 2019, Tracking Fe mobility and Fe speciation in subduction zone fluids at the slab-mantle interface in a subduction channel: A tale of whiteschist from the Western Alps: Geochimica et Cosmochimica Acta, v. 267, p. 1-16.

Ferry, J. M., and Watson, E. B., 2007, New thermodynamic models and revised calibrations for the Ti-in-zircon and Zr-in-rutile thermometers: Contributions to Mineralogy and Petrology, v. 154, no. 4, p. 429-437.

Gong, B., Zheng, Y.-F., and Chen, R.-X., 2007, TC/EA-MS online determination of hydrogen isotope composition and water concentration in eclogitic garnet: Physics and Chemistry of Minerals, v. 34, no. 10, p. 687-698.

Li, X.-H., Liu, Y., Li, Q.-L., Guo, C.-H., and Chamberlain, K. R., 2009, Precise determination of Phanerozoic zircon Pb/Pb age by multicollector SIMS without external standardization: Geochemistry, Geophysics, Geosystems, v. 10, no. 4.

Li, X.H., Tang, G., Gong, B., Yang, Y., Hou, K., Hu, Z., Li, Q., Liu, Y., and Li, W., 2013, Qinghu zircon: A working reference for microbeam analysis of U-Pb age and Hf and O isotopes: Chinese Science Bulletin, v. 58, no. 36, p. 4647-4654.

Liu, Y.S., Gao, S., Hu, Z., Gao, C., Zong, K., and Wang, D., 2009, Continental and Oceanic Crust Recycling-induced Melt–Peridotite Interactions in the Trans-North China Orogen: U–Pb Dating, Hf Isotopes and Trace Elements in Zircons from Mantle Xenoliths: Journal of Petrology, v. 51, no. 1-2, p. 537-571.

Liu, Y.S., Hu, Z., Gao, S., Günther, D., Xu, J., Gao, C., and Chen, H., 2008, In situ analysis of major and trace elements of anhydrous minerals by LA-ICP-MS without applying an internal standard: Chemical Geology, v. 257, no. 1, p. 34-43.

Ludwig, K.R., 2003. ISOPLOT 3.00: A Geochronological Toolkit for Microsoft Excel. Berkeley Geochronology Center, California, Berkeley.

Sláma, J., Košler, J., Condon, D. J., Crowley, J. L., Gerdes, A., Hanchar, J. M., Horstwood, M. S. A., Morris, G. A., Nasdala, L., Norberg, N., Schaltegger, U., Schoene, B., Tubrett, M. N., and Whitehouse, M. J., 2008, Plešovice zircon — A new natural reference material for U–Pb and Hf isotopic microanalysis: Chemical Geology, v. 249, no. 1, p. 1-35.

Stacey, J. S., and Kramers, J. D., 1975, Approximation of terrestrial lead isotope evolution by a two-stage model: Earth and Planetary Science Letters, v. 26, no. 2, p. 207-221.

Zheng, Y. F., Fu, B., Li, Y. L., Xiao, Y. L., and Li, S. G., 1998, Oxygen and hydrogen isotope geochemistry of ultrahigh-pressure eclogites from the Dabie Mountains and the Sulu terrane: Earth and Planetary Science Letters, v. 155, no. 1-2, p. 113-129.

Zheng, Y. F., Wang, Z. B., Li, S. G., and Zhao, Z. F., 2002, Oxygen isotope equilibrium between eclogite minerals and its constraints on mineral Sm-Nd chronometer: Geochimica et Cosmochimica Acta, v. 66, no. 4, p. 625-634.

Zheng, Y.-F., 1993a, Calculation of oxygen isotope fractionation in anhydrous silicate minerals: Geochimica et Cosmochimica Acta, v. 57, no. 5, p. 1079-1091.

Zheng, Y.-F., 1993b, Calculation of oxygen isotope fractionation in hydroxyl-bearing silicates: Earth and Planetary Science Letters, v. 120, no. 3, p. 247-263.
